# The kinase Rio1 and a ribosome collision-dependent decay pathway survey the integrity of 18S rRNA cleavage

**DOI:** 10.1371/journal.pbio.3001767

**Published:** 2024-04-25

**Authors:** Melissa D. Parker, Elise S. Brunk, Adam J. Getzler, Katrin Karbstein

**Affiliations:** 1 The Skaggs Graduate School of Chemical and Biological Sciences, The Scripps Research Institute, La Jolla, California, United States of America; 2 The Herbert Wertheim UF Scripps Institute for Biomedical Innovation and Technology, Jupiter, Florida, United States of America; Yale University, UNITED STATES

## Abstract

The 18S rRNA sequence is highly conserved, particularly at its 3′-end, which is formed by the endonuclease Nob1. How Nob1 identifies its target sequence is not known, and in vitro experiments have shown Nob1 to be error-prone. Moreover, the sequence around the 3′-end is degenerate with similar sites nearby. Here, we used yeast genetics, biochemistry, and next-generation sequencing to investigate a role for the ATPase Rio1 in monitoring the accuracy of the 18S rRNA 3′-end. We demonstrate that Nob1 can miscleave its rRNA substrate and that miscleaved rRNA accumulates upon bypassing the Rio1-mediated quality control (QC) step, but not in healthy cells with intact QC mechanisms. Mechanistically, we show that Rio1 binding to miscleaved rRNA is weaker than its binding to accurately processed 18S rRNA. Accordingly, excess Rio1 results in accumulation of miscleaved rRNA. Ribosomes containing miscleaved rRNA can translate, albeit more slowly, thereby inviting collisions with trailing ribosomes. These collisions result in degradation of the defective ribosomes utilizing parts of the machinery for mRNA QC. Altogether, the data support a model in which Rio1 inspects the 3′-end of the nascent 18S rRNA to prevent miscleaved 18S rRNA-containing ribosomes from erroneously engaging in translation, where they induce ribosome collisions. The data also demonstrate how ribosome collisions purify cells of altered ribosomes with different functionalities, with important implications for the concept of ribosome heterogeneity.

## Abstract 2

A previous study in PLOS Biology found that the kinases Rio1, Nob1 and Pno1 cooperate to establish a checkpoint that prevents the escape of immature ribosomes into polysomes. This Update Article shows that Rio1 only releases accurately processed rRNAs into the translating pool and that misprocessed rRNAs are removed via a ribosome collision-dependent mechanism.

## Introduction

Ribosomes are the molecular machines responsible for protein synthesis in all cells. Maintaining translation fidelity and ensuring protein homeostasis requires proper ribosome assembly, which involves the transcription of 4 ribosomal RNAs (rRNAs), coupled to pre-rRNA processing, folding, and binding to 79 ribosomal proteins (RPs) in a series of ordered steps involving over 200 transiently binding assembly factors [1,2]. During the final cytoplasmic assembly steps of the small ribosomal subunit (40S), cells have established a series of quality control (QC) mechanisms regulated by assembly and translation factors to probe the structural integrity and function of nascent ribosomes [3–7]. These QC checkpoints are important for maintaining healthy cells, as cancer cells contain mutations that bypass ribosome QC [6–9] or have altered RP stoichiometry leading to ribosome heterogeneity [10–12]. In addition to reducing ribosome abundance, haploinsufficiency of RPs can result in misassembled ribosomes lacking these RPs and predisposes patients to cancer [13–19].

In the final stages of 40S ribosome assembly in yeast, the 18S rRNA 3′-end is formed from the precursor 20S rRNA by the essential endonuclease Nob1 [20–24], promoted by its binding partner Pno1 [25]. Immediately prior to Nob1-mediated 18S rRNA cleavage, the precursor of the 40S (pre-40S) subunit containing 20S rRNA is bound to 2 assembly factors, Nob1 and Pno1 [7,26–31]. This intermediate also lacks the RP Rps26, whose binding site is blocked by Pno1 [30,32–34]. Pno1 stabilizes Nob1 on the ribosome and Nob1 blocks mRNA recruitment, thus creating a QC checkpoint that blocks pre-40S from translation [7,25]. After Nob1-dependent 18S rRNA cleavage, the ATPase Rio1 removes both Nob1 and Pno1 from the nascent 40S subunit, allowing for the recruitment of mRNA and Rps26 [7,30,31]. Therefore, Rio1 is responsible for monitoring whether 18S rRNA 3′-end cleavage has occurred, only licensing ribosomes with mature 18S rRNA for translation [7].

It is vital for cells to block these immature pre-40S ribosomes from participating in translation, as translating 20S pre-rRNA-containing pre-40S ribosomes have reduced translational fidelity and do not support cell growth [3,7,35]. Interestingly, 18S rRNAs with as few as 3 nucleotides of precursor rRNA sequence retained at the 18S rRNA 3′-end do not support cell viability in yeast either [36], suggesting that not only is cleavage important, but that it must be precise. However, Nob1 does not always identify the cleavage site correctly, as Nob1 frequently miscleaves its rRNA substrate in vitro [5,22–24]. How Nob1 recognizes its cleavage site remains unknown, as does whether Nob1 miscleaves endogenous 18S rRNA in vivo, whether cleavage accuracy affects ribosome function, and if so, whether cleavage accuracy is monitored to prevent miscleaved rRNA-containing ribosomes from translating.

In addition to QC during ribosome assembly, cells actively monitor translation, targeting aberrant mRNA, rRNA, and nascent peptides for degradation [37–41]. For example, when a mutation in the 18S rRNA decoding site (18S:A1492C) renders the 40S ribosome unable to bind and decode incoming tRNA during translation [42,43], this mutant 18S rRNA is degraded through the so-called 18S nonfunctional rRNA decay (18S NRD) pathway [44,45] (S1A Fig). In this pathway, the defective ribosomes, which likely stall at the initiation site, due to their inability to bind tRNA, are recognized by the ubiquitin E3 ligases Mag2 and Hel2, leading to ubiquitination of Rps3 (uS3, [46–48]). This might involve collisions with a scanning ribosome [49]. The stalled initiation complexes are then split by Dom34 and/or the Rqt complex (Rqt2, Rqt3, Rqt4), ultimately leading to Xrn1-dependent decay of the aberrant 18S rRNA [45,50].

Ribosome collisions are also the initiating events in mRNA QC (S1B Fig, [51,52]). mRNAs that are damaged [53–55] or contain certain “stall-sequences” [56–58] trap ribosomes at these locations. Ultimately, this leads to collisions with the trailing ribosome, which are mediated via a unique 40S/40S interface, which includes the nonessential ribosomal protein Asc1 [52,59,60]. These collided disomes are then recognized by Mbf1, which prevents frameshifting [61,62], and the ubiquitin-ligase Hel2, which ubiquitinylates Rps20 (uS10) [60,63]. Additional components block new translation [64] and lead to decapping and ultimately Xrn1-mediated decay of the mRNA [65]. Moreover, the stall can be resolved via one of 2 ways: in the predominant pathway, which we term RQC (ribosome-associated quality control) here, the Rqt complex splits the stalled ribosome into 40S and 60S, likely in an iterative process until all ribosomes have been cleared. Alternatively, in the less-predominate pathway, the endonuclease Cue2 can cleave the mRNA between the stalled and trailing ribosomes [65], opening a binding site for Xrn1-mediated decay of the 3′-end of the mRNA. In addition, Dom34 splits the trailing ribosome into 40S and 60S subunits [66]. This pathway is referred to as no-go-decay (NGD).

Using next-generation sequencing, yeast genetics, and biochemical techniques, we show that miscleaved 18S rRNAs are formed in vivo, but that their fate is regulated by the Rio1-mediated QC step and a collision-dependent decay pathway. Next-generation sequencing of the 3′-ends of 18S rRNAs from wild-type yeast cells show that at the steady state about 2% of 18S rRNAs are miscleaved. Biochemical and genetic data demonstrate that truncated, miscleaved 18S rRNAs, even at these low concentrations, disrupt cell growth, because they engage in translation where their slower elongation leads to ribosome collisions with trailing correctly matured rRNAs. The resulting complexes are targeted for ribosome decay involving the proteins Asc1, Hel2, and Xrn1. In contrast, Mag2-dependent ubiquitinylation of Rps3 (uS3) is not required. We show that miscleaved 18S rRNAs are increased in abundance not just when components of the collision-mediated RNA-decay machinery are deleted, but also upon bypassing the Rio1-mediated QC step, implicating Rio1 in QC of correct Nob1 cleavage. Confirming this, ribosomes containing truncated, miscleaved 18S rRNAs retain Pno1, indicating that they failed to pass the Rio1-mediated checkpoint. Finally, Rio1 has a stronger binding affinity for 18S rRNAs with correct 3′-ends than miscleaved 3′-ends. Altogether, these data support a model in which Rio1 inspects the 3′-end of 18S rRNA, ensuring only ribosomes with accurately cleaved 18S rRNA are released into the translating pool. On the other hand, Rio1 will not remove Nob1 or Pno1 from ribosomes containing miscleaved 18S rRNA, thus restricting their translation. The data also demonstrate how dysfunctional ribosomes produced via leaky checkpoints are removed from the translating pool, thereby purifying cells of dysfunctional heterogenous ribosomes.

## Results

### Nob1 miscleaves pre-18S rRNA

The 3′-end of 18S rRNA is highly conserved and identical in organisms ranging from yeast to humans (S2A Fig), indicating the importance of this rRNA segment. Furthermore, maintaining faithful 18S rRNA cleavage during ribosome assembly is critical, as 18S rRNAs retaining as few as 3 additional nucleotides (nts) at their 3′-end do not support viability in S. *cerevisiae* [36]. While important, accurate cleavage of the 3′-end of 18S rRNA during pre-rRNA processing may not be a simple task. Nob1 cleaves the premature (pre-) rRNA between 2 adenosines, and there are multiple pairs of adenosines nearby (S2B Fig) that Nob1 could potentially recognize and cleave. Consistently, in vitro experiments have shown that Nob1 frequently miscleaves its 18S rRNA substrate, resulting in multiple cleavage products [5,22–24]. What remains unknown is whether Nob1 (alone or in combination with other assembly factors) can identify and accurately cleave endogenous 18S rRNA in vivo.

**Fig 1:**
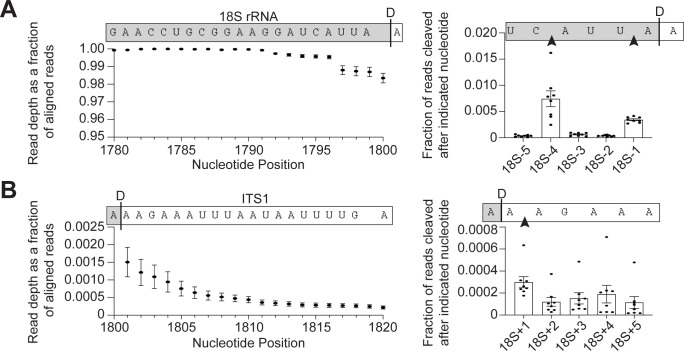
Miscleaved 18S rRNA are rare in vivo. (A, B) 3′-RACE-sequencing of 18S rRNA extracted from 40S subunits purified from Gal::Pno1;Gal::Dim1 cells grown in glucose to deplete endogenous Pno1 and Dim1 and supplemented with plasmids encoding Pno1 and Dim1-E85A, an inactive mutant that prevents dimethylation of 18S rRNA [69]. Left: Read depth at each nucleotide normalized to the number of reads aligning to the 3′-end of 18S rRNA. Nucleotide positions in 18S rRNA are indicated. Above each graph is a schematic of the 18S rRNA and the ITS1 sequence above their corresponding nucleotide position and read depth. A black line labeled “D”" to indicate the D-cleavage site at the canonical 3’-end of 18S rRNA indicates the 3′-end of 18S rRNA. Read depth over the 21 nucleotides at the 3′-end of 18S rRNA (A) and the 20 nucleotides at the 5′ end of ITS1 (B). Right: The fraction of reads miscleaved after each of the final 5 nucleotides of 18S rRNA (A) or after each of the first 5 nucleotides in ITS1 (B) surrounding the canonical 3′-end of 18S rRNA. Data are the average of 8 biological replicates, and error bars indicate standard error of the mean (SEM) (error bars are too small to be seen for many data points). Raw sequencing data are available via the GEO database under accession number GSE259239. Processed data to make the panels are available as Supporting information under **S1 Data**. ITS1, Internal Transcribed Spacer 1; rRNA, ribosomal RNA.

To examine the accuracy of 18S 3′-end formation, we performed 3′-RACE (rapid amplification of cDNA ends) sequencing on 18S rRNA to survey the 3′-ends of 18S rRNA. We grew yeast to early stationary phase (between OD_600_ 1.2 to 1.8), purified the 40S ribosomal subunits, and extracted the 18S rRNA. Next, a linker was ligated to the 3′-ends of the RNA to protect it from degradation and accurately identify the 3′-ends. This linker was used to prime reverse transcription, creating cDNAs that were subsequently converted into sequencing libraries using 18S rRNA-specific primers for analysis of 18S rRNA 3′-ends. Due to concerns that reverse transcription through the Dim1-dimethylation site in 18S rRNA (m^6^_2_A1781 and m^6^_2_A1782) would pose complications [67,68], we used a yeast strain containing a mutation in Dim1 (Dim1-E85A) that does not dimethylate 18S rRNA [69], thus allowing us to sequence the final 40 to 60 nucleotides of 18S rRNA. Control experiments indicate that this does not affect the frequency of miscleavage (S2C Fig). We obtained 2.8 to 4 million reads per sample, with 96% to 99% of reads aligning to the 3′-end of 18S rRNA. Mapping these reads showed that approximately 98% of the stable 18S rRNAs terminate at the mature 3′-end (Figs 1A and S2B). Only a small percentage (approximately 2%) of RNAs are miscleaved, producing mostly slightly shortened and some lengthened 18S rRNA products. The most frequent (0.03%) miscleavage downstream of the canonical 3′-end occurred between 2 adenosines one nucleotide into Internal Transcribed Spacer 1 (ITS1), the precursor rRNA sequence 3′ to 18S rRNA (18S+1 nt), while the most abundant (0.8%) upstream miscleavage occurred between a cytidine and an adenosine 4 nucleotides upstream of site D (18S-4 nts, Fig 1). Thus, these data indicate a propensity for Nob1 to cleave 5′ to an adenosine but show that most 18S rRNAs have a correctly formed 3′-end.

### Cell growth is perturbed upon expression of miscleaved rRNAs

Above, we observed that at the steady state miscleaved 18S rRNAs are rare in vivo. However, previous in vitro data indicated that Nob1 frequently miscleaves RNA [5,22–24]. This discrepancy led us to hypothesize that additional mechanisms might exist in vivo to eliminate miscleaved 18S rRNAs. This would be important if miscleaved 18S rRNA have a detrimental effect on cellular fitness. To determine whether ribosomes containing miscleaved 18S rRNA have a negative impact on cell growth, we took advantage of a temperature sensitive *S*. *cerevisiae* strain (NOY504) containing a deletion of a nonessential subunit of RNA polymerase I, RPA12 (RRN4) [70]. At nonpermissive temperatures, this mutation reduces RNA polymerase I (PolI) complex stability, thus limiting PolI transcription to less than 5% of that at permissive temperatures [71]. NOY504 cells were transformed with plasmids encoding an RNA polymerase II promoter-driven 35S rDNA, which encodes the 18S rRNA, 5.8S rRNA, and 25S rRNA ([72], S2D Fig). This plasmid-encoded rRNA can be distinguished from endogenous rRNA by a sequence tag in 18S rRNA (Gal7 promoter constructs) or by sequence tags in both 18S and 25S rRNA (GPD promoter constructs), which are functionally neutral [36,73–75]. Therefore, at the permissive temperature (30°C), cells express both endogenous and plasmid-encoded 18S rRNA, 5.8S rRNA, and 25S rRNA, with endogenous rRNA in vast excess [44]. However, at 37°C, the endogenous rRNA is not transcribed and the cells rely solely on the plasmid-encoded rRNA transcribed by RNA polymerase II.

**Fig 2:**
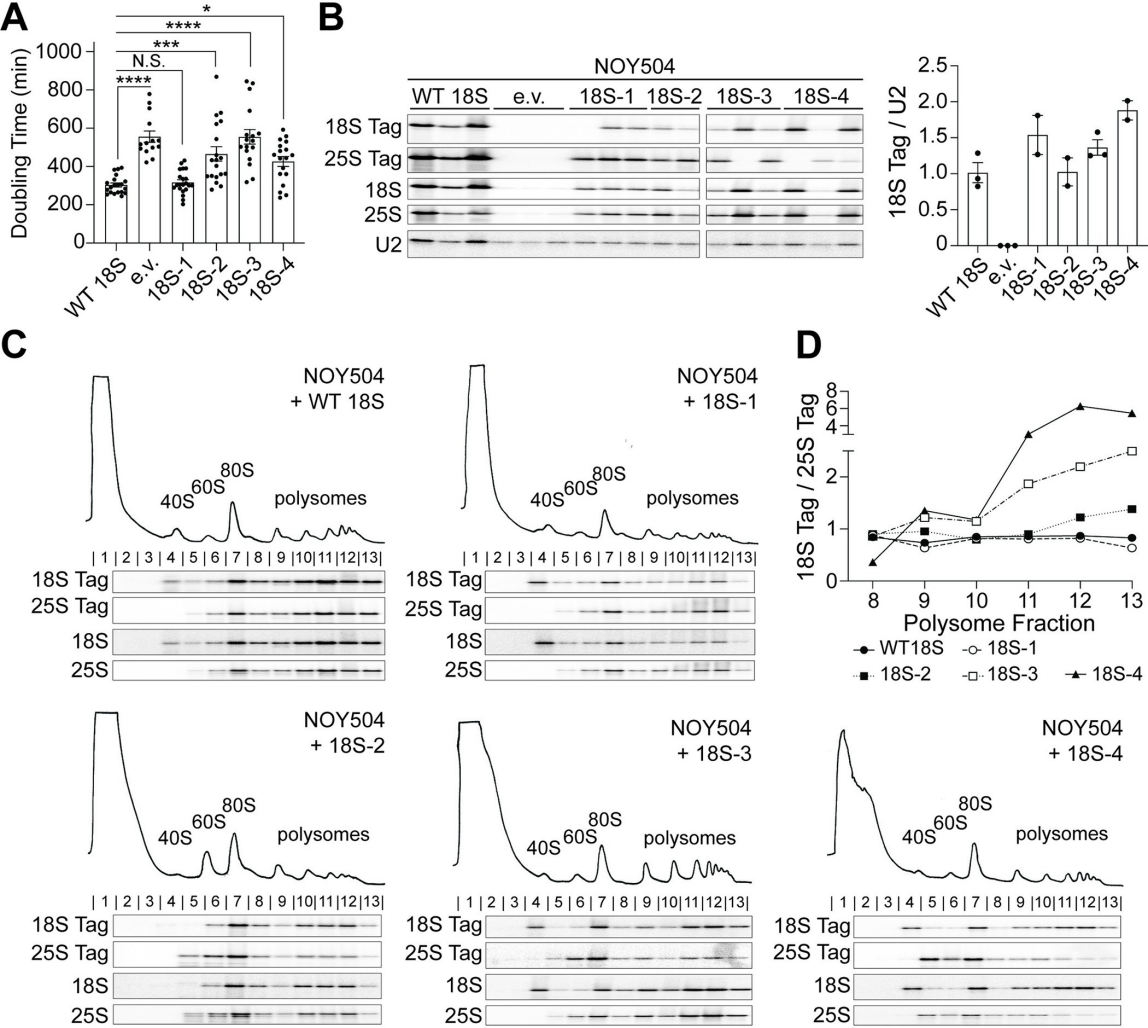
By themselves, miscleaved ribosomes are partially functional but stable. (A) Doubling times of NOY504 cells depleted of endogenous rRNA via growth at 37°C and expressing plasmid-encoded WT 18S rRNA (GPD promoter), miscleaved 18S rRNAs, or an empty vector (e.v.). Data are the averages of 14–21 biological replicates, and error bars indicate SEM. N.S., not statistically significant, * p_adj_ < 0.05, *** p_adj_ < 0.001, **** p_adj_ < 0.0001, by one-way ANOVA (Dunnett’s multiple comparisons test). (B) Left: Northern blot of total RNA from cells in panel A. Plasmid-encoded 18S rRNA and 25S rRNA each contain a neutral, unique sequence that is not present in endogenous rRNAs and was specifically detected with a northern probe (18S Tag and 25S Tag, respectively, [36]). Additional probes were used to detect all 18S or 25S rRNAs using sequences common to the plasmid and endogenous rRNAs (18S and 25S, respectively). All samples were run on the same northern blot and the order was edited for clarity. Right: Levels of plasmid-encoded 18S rRNAs were normalized to U2 snRNA. Data are the averages of 2 to 3 biological replicates, and error bars indicate SEM. As previously observed, cells encoding mutant 18S rRNAs are under selective pressure and can undergo homologous recombination with the endogenous 18S rDNA, resulting in a loss of the 18S rRNA tag and the miscleavage phenotype [44]. We have therefore excluded such replicates from quantification. (C) Northern blots of 10%–50% sucrose gradients from lysates of cells in panel A. Northern blots were probed for plasmid-encoded 18S and 25S rRNAs (Tag), as well as all 18S and 25S rRNAs. Fraction numbers are listed above the northern blots. (D) Quantification of the plasmid-encoded 18S rRNA (18S Tag) levels normalized to the plasmid-encoded 25S rRNA (25S Tag) in each polysome fraction (fractions 8–13) from panel C. Raw numerical values to make panels A, B, and D are available as Supporting information under **S2 Data**. rRNA, ribosomal RNA; WT, wild type.

To delineate the effects from rRNA miscleavage on cell viability and translation, we encoded truncated 18S rRNA on the plasmid, thereby rendering all plasmid-encoded rRNA “miscleaved” (S2D Fig). We were unable to test the effects of elongated 18S rRNA, miscleaved within ITS1, on cell viability because this required the expression of the rRNAs on 2 separate plasmids (one containing the miscleaved, elongated 18S rDNA template and a second plasmid containing the 5.8S and 25S rDNAs), and cells expressing rRNA from 2 plasmids grew too slowly to be measured reliably in our system. However, as indicated above, previous data demonstrate that rRNAs retaining 3 extra nucleotides do not support cell growth [36]. We first compared the growth of NOY504 cells expressing wild type (WT) or miscleaved, truncated rRNA variants by measuring their doubling times at nonpermissive temperature in a continuous growth assay. While shortening the rRNA by 1 nucleotide does not result in any growth defects, cells expressing rRNAs mimicking miscleavage 2 to 4 nucleotides upstream of the canonical cleavage site produced 1.5- to 2-fold slower growth compared to cells expressing WT 18S rRNA, nearly as slow, or as slow as cells lacking plasmid-encoded rRNA entirely (Fig 2A). Importantly, both WT and miscleaved 18S rRNAs produce similar amounts of plasmid-derived tagged 18S rRNA (Fig 2B), demonstrating that the growth defects cannot be explained by reduced amounts of rRNA. Moreover, these miscleaved RNAs are actively translating, as gradient sedimentation shows that both correctly cleaved and miscleaved tagged 18S rRNA are found throughout the polysomes (Fig 2C). Quantification reveals that nonfunctional miscleaved 18S-tagged ribosomes accumulate within polysomes (Fig 2D), consistent with slower elongation of these partially functional ribosomes. Thus, miscleaved 18S rRNAs can translate mRNAs but are defective, leading to substantial growth defects.

In addition, miscleaved rRNAs do not accumulate rRNA precursors (Fig 2B), strongly suggesting that Nob1 may only recognize the 3′-A, which is common to all miscleaved substrates and thus lacks strong sequence specificity. This observation, together with the frequent miscleavage observed in vitro [5,22–24], raises the question of how the uniformity of the 18S rRNA 3′-end that we observe in WT cells is achieved.

### Miscleaved rRNAs are destabilized by correctly cleaved rRNAs

**Fig 3:**
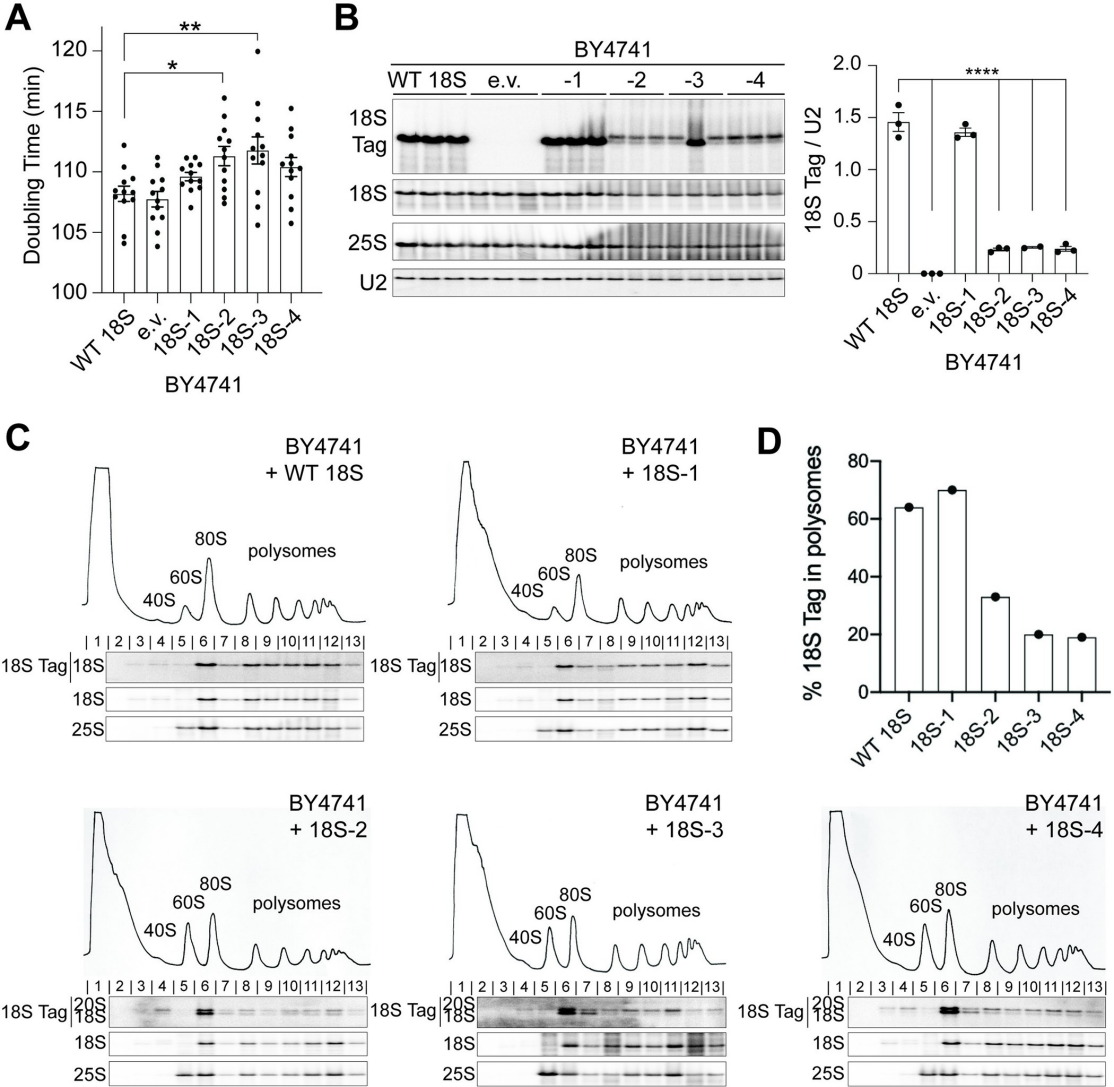
Miscleaved 18S rRNAs perturb the translation of correctly matured 40S. (A) Doubling times of BY4741 cells expressing both endogenous rRNAs and plasmid-encoded 18S rRNAs (Gal7 promoter) or an empty vector (e.v.) grown at 30°C. Data are the averages of 12 biological replicates, and error bars indicate SEM. *p_adj_ < 0.05, **p_adj_ < 0.01 by one-way ANOVA (Dunnett’s multiple comparisons test). All other differences in doubling time were not statistically significant. (B) Left: Northern blot of total RNA from cells in panel A. Right: Plasmid-encoded 18S rRNA accumulation was normalized to U2 snRNA. Data are the averages of 2 to 3 biological replicates, and error bars indicate SEM. The second replicate of 18S-3 was excluded from analysis as explained in the legend of Fig 2. **** p_adj_ < 0.0001, by one-way ANOVA (Dunnett’s multiple comparisons test). The difference in 18S Tag/U2 accumulation between WT 18S and 18S-1 was not statistically significant. (C) 10%–50% sucrose gradients of lysates from cells in panel A. Below the absorbance profile at 254 nm are northern blots of the plasmid-encoded 18S rRNA (Tag), as well as total 18S and 25S rRNAs. (D) The fraction of plasmid-encoded rRNA in polysomes (fractions 8–13) was quantified from panels C. Raw numerical values to make panels A, B, and D are available as Supporting information under **S3 Data**. rRNA, ribosomal RNA; WT, wild type.

In the above experiments, we utilized a system in which most 18S rRNA is miscleaved. However, as also shown above, in a physiologic miscleavage context, cells produce only a small amount of miscleaved 18S rRNA alongside mostly correctly cleaved 18S rRNAs. To mimic this, we measured the growth of a WT *S*. *cerevisiae* strain (BY4741) with fully functional RNA polymerase I, expressing the same plasmid-encoded WT or truncated, miscleaved 18S rRNAs. Surprisingly, expression of plasmid-encoded 18S rRNAs miscleaved 2 or 3 nucleotides upstream of the mature 18S 3′-end caused small, but significant dominant-negative growth defects, despite being vastly outnumbered by endogenous WT ribosomes. The 18S-4 mutant also caused a slight, although nonsignificant growth defect (Fig 3A). Thus, in the presence of correctly matured rRNA, miscleaved 18S rRNAs, even in small amounts, perturb the ability of cells to grow, suggesting that translating ribosomes containing miscleaved 18S rRNAs disrupt translation by subunits containing correctly cleaved 18S rRNA.

Moreover, the same plasmid-encoded miscleaved rRNAs that accumulated to WT levels when they are the only rRNAs in the cell (Fig 2B), are reduced 5-fold relative to correctly cleaved wt 18S rRNA when expressed in a background of normal correctly cleaved ribosomes (in the BY4741 background, Fig 3B). Importantly, this only happens to the dysfunctional −2, −3, and −4 miscleaved 18S rRNAs, not the plasmid-encoded wt 18S rRNA or the functional −1 miscleaved rRNA, which does not demonstrate growth defects.

Since miscleaved 18S rRNAs were encoded on the same Gal7-driven plasmid as the WT 18S rRNA, it was unlikely that transcriptional differences between the 2 strains would explain the observation that miscleaved 18S rRNA was less abundant than WT 18S rRNA in the BY4741 background while equally abundant in the NOY504 background. Instead, this observation indicates that the WT and miscleaved rRNAs are differentially stable. To test this directly, we attempted to measure turnover of the plasmid-encoded WT 18S rRNA, or miscleaved 18S rRNA in the background of endogenous 18S rRNA, using a pulse-chase experiment. Transcription of tagged, plasmid-encoded rRNA was induced by addition of galactose to the Gal7-driven plasmids, and then turned off by addition of glucose. Cells were harvested at different time intervals, and total rRNA was isolated and analyzed using northern blotting. These data demonstrate that while tagged WT 18S rRNA is stable throughout the experiment (and only diluted by cell division), the miscleaved rRNAs are degraded almost entirely in less than 2 h (S3A Fig). However, these experiments are complicated by the fact that for WT plasmids, decay of 18S rRNA is measured directly, while for the miscleaved mutant rRNAs, 18S rRNA hardly accumulates (Figs 3B and S3A). Thus, for the miscleaved rRNAs, the observed rate constant is a combination of the rate constant for maturation of the 20S rRNA and its decay [76]. Notably, the data in Fig 2B demonstrate that maturation is not affected by these mutations, suggesting that the overall faster decay of the mutant rRNAs is driven by much faster decay once the 18S rRNA is formed.

To further confirm the conclusion that reduced steady-state levels of dysfunctional −2, −3, and −4 miscleaved 18S rRNAs relative to WT 18S rRNA or the functional −1 miscleaved rRNA were due to differences in decay rates and not due to differences in transcription rates, we uncoupled the changes in rRNA abundance from changes in transcription. This was done by measuring the levels of plasmid encoded wt or miscleaved 18S-2 rRNA, encoded from either the GPD or the Gal7-promoter-driven plasmids (grown in glucose or galactose, respectively), in NOY504 cells which were switched from 30 to 37°C. By switching the growth temperature, transcription of genomically encoded rRNA is turned off. Thus, over time the genomically encoded rRNAs will disappear (diluted via cell division), and the plasmid-encoded rRNAs will start making up a larger and larger fraction of the total RNA. Because it is the presence or absence of genomically encoded rRNAs that leads to the difference in the levels of the dysfunctional miscleaved 18S rRNAs (Fig 2A versus Fig 3A), the dysfunctional rRNA should become more stable as the genomically encoded rRNA disappears. Because any changes in transcription arising from the temperature shift would occur rapidly after the shift, while changes in rRNA occur slowly after many cell divisions, this experiment uncouples changes in transcription from changes in decay. Indeed, the data in S3B and S3C Fig show that levels of miscleaved 18S-2 rise about 4 doublings (>12 h) after the temperature switch, as the genomic rRNAs disappear. Thus, the difference in accumulation of the same dysfunctional miscleaved rRNAs in the presence or absence of genomically encoded WT RNAs is not due to differences in transcription and must therefore arise from faster decay due to the presence or absence of ribosomes containing genomically encoded WT 18S rRNAs.

Finally, we tested whether the tagged 18S rRNA was uniformly depleted from cells, or specifically from translating ribosomes, by measuring its distribution over a polysome gradient. Importantly, the data in Fig 3C demonstrate that dysfunctional miscleaved rRNAs, but not the functional WT or −1 miscleaved rRNAs, are depleted from the polysomes when they are expressed around correctly cleaved rRNAs (Fig 3C). Again, this observation contrasts with the finding that the same rRNAs are enriched in the polysomes when miscleaved 18S rRNA are the only rRNAs present (Fig 2C and 2D).

Thus, taken together, the miscleaved plasmid-encoded rRNAs are functionally defective, leading to dominant-negative growth defects. Moreover, while the ribosomes containing these miscleaved rRNAs are stable and able to translate when all ribosomes are functionally defective, mixing them in with functional ribosomes depletes the miscleaved rRNA-containing ribosomes from the polysomes and renders them unstable.

### Ribosomes with defective miscleaved 18S rRNA translate more slowly

The simplest model from the dominant-negative growth defect that arises from miscleaved rRNAs is that the miscleaved ribosomes perturb translation of all ribosomes by slowing or stalling on mRNA, which would lead to a collision with the next ribosome. Stalled ribosomes lead to ribosome collisions, which result in the decay of the translated mRNA via NGD or mRNA quality control (RQC), the degradation of the nascent peptide chain through ribosome-associated quality control. Moreover, ribosomes containing nonfunctional 18S rRNA, which cannot bind tRNAs and are presumably stalled at the start-site, are targeted by 18S nonfunctional rRNA decay (NRD) [37–41]. We thus hypothesized that the miscleaved but partially functional ribosomes translate, albeit more slowly, ultimately allowing for collisions with the subsequent ribosome. This would explain their depletion from the polysomes only when functional rRNAs are around, as collisions would not occur if all ribosomes are equally defective.

**Fig 4:**
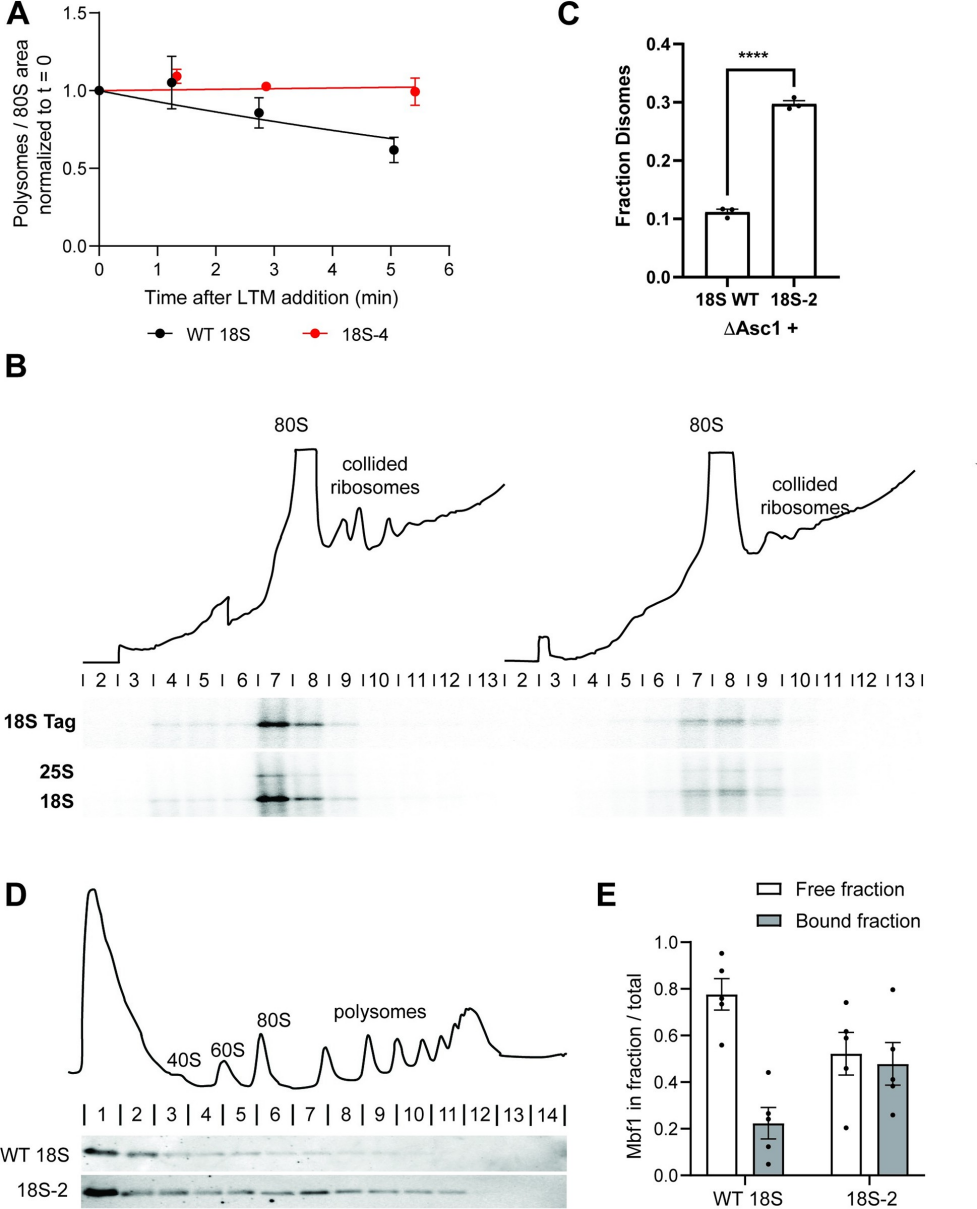
Miscleaved 18S rRNAs translate slowly and lead to ribosome collisions. (A) Ribosome run-off experiment to measure translation speed. Polysome disappearance in NOY504 cells expressing plasmids encoding WT 18S rRNA or the miscleaved 18S-4 rRNA was quantified using sucrose gradient analysis after blocking translation initiation with LTM. The polysome area was plotted relative to 80S ribosomes as a function of time after LTM addition. Data were fit to a single exponential decay model and gave rate constants for ribosome run-off of 0.07 and −0.004 min^-1^ for WT and 18S-4 cells, respectively. (B) Sucrose gradient profile of MS2-tagged, RNA affinity-purified, plasmid-encoded ribosomes purified from ΔAsc1 cells (top). The positions of monosomes and disomes are indicated, and the northern blots from RNAs extracted from each fraction is displayed below. (C) Quantification of 18S TAG RNA normalized to 18S rRNA (to account for loading differences) from the northern blot in B and 2 biological replicates. ****p_adj_ < 0.0001, by unpaired *t* test. (D) Sucrose gradient profile of cells expressing WT 18S (top), and anti-HA western blot from these and the corresponding 18S-2 cells (bottom). (E) Quantification of western blots for Mbf1-HA over sucrose gradients in BY4741 cells expressing either WT or 18–2 rRNA in the background of endogenous WT ribosomes. The graph is a quantification of 5 biological replicates. Raw numerical values to make panels A, C, and D are available as Supporting information under **S4 Data**. LTM, lactimidomycin; rRNA, ribosomal RNA; WT, wild type.

To test this model, we verified whether the miscleaved ribosomes translate more slowly (and therefore could cause collisions with endogenous WT ribosomes), using ribosome run-off assays [77]. In these experiments, we blocked translation initiation via the addition of lactimidomycin (LTM). We then harvested cells at different time points after LTM addition to allow run-off of ribosomes from the mRNAs on which translation had been initiated prior to addition of the drug. Ribosome binding to RNAs was assessed by polysome profiling and quantification of the area of the 80S peak (run-off) and the polysomes. The data in Figs 4A and S4 demonstrate that indeed ribosomes from cells encoding only WT 18S rRNA run off their mRNAs more quickly than ribosomes from cells encoding only miscleaved 18S-4 rRNA. This finding demonstrates that indeed ribosomes with miscleaved 18S rRNA translate more slowly, consistent with their accumulation in polysomes, when they are by themselves (Fig 2D).

### Ribosomes with defective miscleaved 18S rRNA form disomes

Next, we wanted to obtain direct evidence for ribosome collisions. To facilitate the detection of collided ribosomes for biochemical and structural studies, many studies of ribosome collisions utilize highly expressed reporters with strong stall sequences [78–82], or anisomycin, an antibiotic that blocks elongation, to induce stalling globally [60,79,82–85]. As desired, this produces large amounts of collisions. In contrast, the defective ribosomes whose potential collisions we are investigating are a minority (approximately 5%) of the total ribosome pool, similar to what one expects under physiological situations [86], rendering their detection via the typically utilized method—RNase digestion of lysates—difficult. To nonetheless demonstrate the accumulation of disomes directly, we therefore first enriched ribosomes containing plasmid-encoded 18S rRNA by affinity purification using the MS2-tag. This approach is inspired by similar experiments from the Inada lab, where collided disomes on the endogenous SDD1 mRNA were purified via the nascent protein chain [58]. In addition, to stabilize disomes against degradation, this experiment was carried out in ΔAsc1 cells. Asc1 is part of the interface of collided disomes [52,59] and is required for the decay of nonfunctional 18S rRNA [48] as well as miscleaved rRNA (see below). The purified, plasmid-derived ribosomes (WT or miscleaved) were then loaded onto a sucrose gradient to separate 40S, 80S, and disomes, and the distribution of plasmid-encoded rRNAs in monosomes and disomes was quantified using northern blotting with the probe for the tag on the plasmid-derived 18S rRNA and normalized using endogenous 18S rRNA to account for loading differences. The data in Fig 4B and 4C show that indeed about 3-fold more disomes are observed in cells containing the miscleaved 18S-2 rRNA relative to cells containing only WT 18S rRNA, providing strong support for the formation of disomes.

### Ribosomes with defective miscleaved 18S rRNA recruit Mbf1

To further confirm the formation of disomes and to start probing their similarity to disomes formed by collisions on damaged mRNAs, we utilized a previously described indirect assay for the formation of collided disomes, the recruitment of Mbf1 [62,85,87]. In unstressed cells Mbf1 is largely unbound to ribosomes. However, under conditions that lead to ribosome collisions, such as the expression of mRNAs with stall sequences, Mbf1 is recruited to collided disomes [62,85,87]. We therefore tested the sedimentation of HA-tagged Mbf1 with ribosomes in cells expressing only WT rRNA (endogenous and plasmid-encoded), or correctly cleaved (endogenous) and plasmid-encoded miscleaved rRNAs. Indeed, while nearly all Mbf1 is free in the cells with only wt 18S rRNA, in the cells with miscleaved 18S rRNA Mbf1 is recruited to ribosomes (Fig 4D), further supporting the formation of collided disomes in cells expression miscleaved rRNAs at physiological concentrations.

Altogether, these data provide strong support for a model that ribosomes containing miscleaved 18S rRNA translate more slowly, therefore inviting collisions with correctly cleaved ribosomes, if these are available (such as in the cells expressing both plasmid-encoded and endogenous WT rRNAs). These collided disomes bind Mbf1 and lead to the degradation of the defective collided ribosomes.

### Collision-mediated decay of miscleaved 18S rRNA requires parts of the RQC machinery

Next, we wanted to know whether the decay of miscleaved partially functional ribosomes required the same factors as decay of defective mRNAs (via RQC or NGD) or nonfunctional rRNA (via NRD). Collided ribosomes form a unique interface involving the 40S RP Asc1 [52,59], which is recognized by the E3 ubiquitin ligase Hel2, which ubiquitinates Rps20 (uS10) [63]. This leads to subunit dissociation either by the RNA helicase Slh1/Rtq2, which together with Rqt3 and Rqt4 is part of the Rqt complex [63], or by Cue2 and Dom34 via NGD (S1B Fig, [65,66]). Degradation of nonfunctional 18S rRNA (NRD) requires Asc1 [48], possibly reflecting collision of scanning 40S subunits with stalled initiation complexes [49], the ubiquitinylation of Rps3 (uS3) by the E3 ligase Mag2 [50], and relies on the cytoplasmic exosome recruitment factor Ski7, the ribosome splitting factors Dom34 and Rqt, and the exonuclease Xrn1 [45,50,59], S1A Fig).

**Fig 5:**
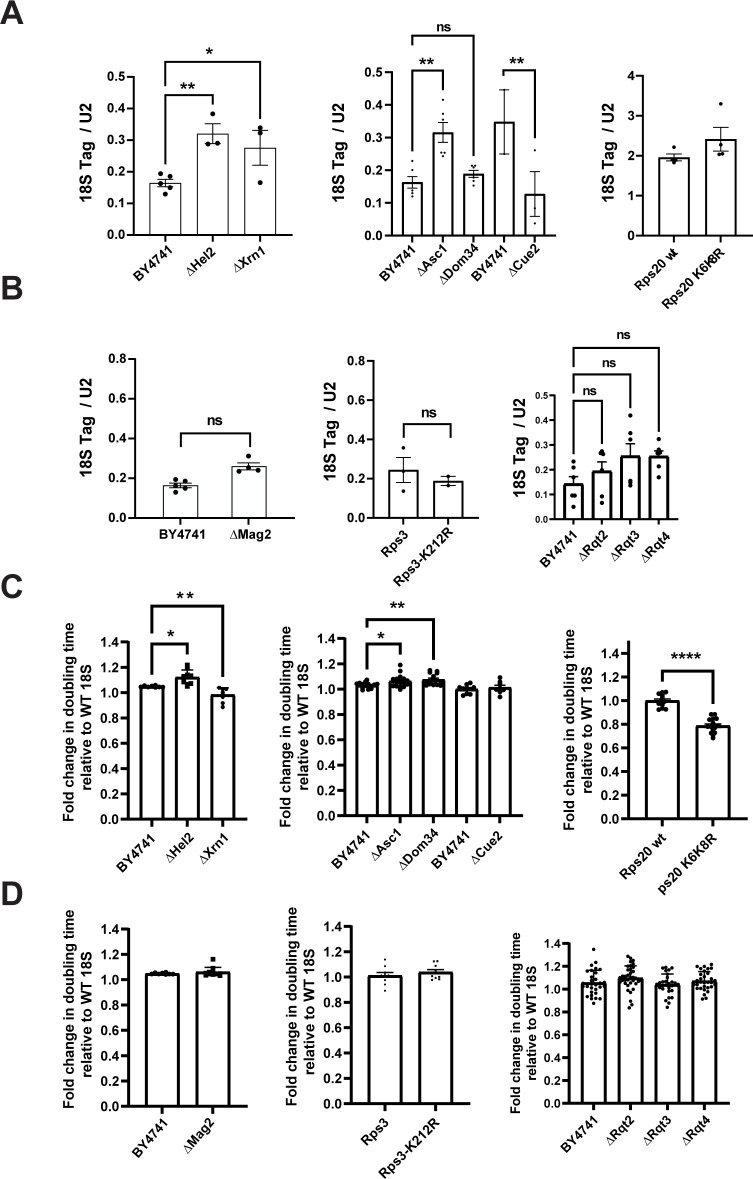
Partial overlap between RQC and decay of dysfunctional 18S rRNA. (A, B) Levels of miscleaved 18S rRNA (18S-2) relative to WT rRNA in cells lacking components of the RQC machinery. Plasmid-encoded 18S rRNA was normalized to U2 snRNA. 18S Tag/U2 ratios from cells expressing 18S-2 were normalized to the 18S/U2 ratios from cells expressing WT 18S for each cell background (fold change = 1). Data are the averages of 2–6 biological replicates, and error bars indicate SEM. N.S., not statistically significant, *p_adj_ < 0.05, **p_adj_ < 0.01, by one-way ANOVA (Dunnett’s multiple comparison’s test) compared to BY4741, or by an unpaired *t* test when WT and mutant Rps20 or Rps3 are compared. (C, D) Changes in doubling time of the indicated cells supplemented with plasmids encoding WT 18S or miscleaved 18S-2 rRNAs grown at 30°C. Except for the experiments with Rps3 and Rps20 mutants, all rRNA plasmids were expressed under the Gal7 promoter. For experiments with Rps3 or Rps20 mutants, 18S rRNA mutants were expressed from the GPD promoter, as Rps3 or Rps20 were under galactose control. ΔAsc1 cells were supplemented with a plasmid encoding U24 snRNA, normally encoded in the *ASC1* intron. Doubling times were normalized to WT 18S for each cell background (fold change = 1). Data are the averages of 12–19 biological replicates, and error bars indicate SEM. N.S. not statistically significant, *p_adj_ < 0.05, **p_adj_ < 0.01, ****p_adj_ < 0.0001 by one-way ANOVA (Dunnett’s multiple comparisons test) compared to WT cells for each 18S rRNA variant. Comparisons not indicated are not statistically significant. Raw numerical values to make this figure are available as Supporting information under **S5 Data**. RQC, ribosome-associated quality control; rRNA, ribosomal RNA; WT, wild type.

We next used northern blotting to ask whether the degradation of the defective partially functional RNAs also required these factors. Deleting Asc1, Hel2, or Xrn1 stabilized plasmid-derived miscleaved 18S rRNA (Figs 5A, S5, S6A and S6B), but not the 20S pre-rRNA (S5A Fig), as expected because most 20S pre-rRNAs are not translating [3]. Moreover, deleting these proteins also affected the dominant growth defects caused by the miscleaved rRNAs (Figs 5C and S5D). Notably, while deleting Asc1 or Hel2 sensitizes cells to miscleaved 18S rRNAs, deletion of Xrn1 partially rescued the dominant-negative growth defect of miscleaved 18S rRNA. Mutation to arginine of K6/K8 in Rps20, which are the target of Hel2-mediated ubiquitination during RQC, stabilizes miscleaved 18S rRNA, albeit moderately (Figs 5A, S5C, and S6C). Consistently, the K6/K8 mutation also affected yeast growth, partially rescuing the dominant-negative growth defect of miscleaved 18S rRNA (Figs 5C and S5E).

In contrast, neither the Rps3_K212R mutation nor deletion of Mag2, the E3 ligase that installs the first ubiquitin on this residue, has an effect on the stability of 18S rRNA (Figs 5B, S5C, S6A and S6F). Consistently, neither of these 2 alterations affected the dominant negative growth defect from the miscleaved rRNAs (Figs 5D and S5).

Deletion of Rqt complex components had no significant effects on stability of the miscleaved rRNA (Figs 5B, S5 and S6E), and also does not affect yeast growth (Figs 5D and S5C), suggesting that the Rqt complex is not required for degradation of miscleaved 18S rRNA.

Deletion of Dom34 and Cue2 has complex effects: while deletion of Dom34 stabilized the 4-nucleotide truncated, miscleaved 18S rRNA, its effect on the 2-nucleotide truncated 18S rRNA is less clear. Notably though, Dom34 is the only gene whose deletion also stabilizes the plasmid-encoded immature 20S pre-rRNA (S5A Fig), as expected because Dom34 is involved in separating 80S-like ribosomes during pre-40S assembly [3]. The resulting accumulation of pre-rRNA and depletion of 18S rRNA [3] would also be expected to mask stabilization of miscleaved 18S rRNA. Indeed, deletion of Dom34 exacerbates the growth defect from the dominant-negative miscleaved 18S rRNAs. Deletion of Cue2 surprisingly destabilized miscleaved 18S rRNA (Fig 5A), potentially suggesting that Cue2 plays a redundant role as it does in mRNA QC [65] and that the alternative decay pathway is more efficient (and utilized in the absence of Cue2). However, deletion of Cue2 has no effect on the dominant-negative growth induced by expression of the miscleaved rRNAs (Fig 5C). This indicates that any role Cue2 might play in decay of miscleaved rRNA is likely minor.

In summary, deletion of Asc1, Hel2, Xrn1, Dom34, and mutation of Rps20-K6/K8 stabilize both the miscleaved 18S rRNA and affect the growth defects arising from the induced expression of these miscleaved rRNAs, thereby strongly supporting the role of these factors in collision-mediated decay of dysfunctional ribosomes. Thus, there are strong parallels between the collision-induced decay of defective mRNAs and rRNAs. Notably, while deletion of Hel2, Asc1, and Dom34 exacerbate the growth effects from miscleaved rRNA, deletion of Xrn1 and mutation of Rps20-K6/K8 partially rescue the dominant growth defects from miscleaved rRNA. We suggest that while all of these factors are required for the degradation of defective rRNA after collisions, the differential effects on the dominant-negative growth defect can be explained because in some cases the resolution of collided disomes is blocked (e.g., deletion of Asc1, Hel2, and Dom34), while in others (mutation of Rps20 and Xrn1), collisions are cleared, but without degrading the defective 18S rRNA. In that model, the collisions themselves would be beneficial, perhaps due to the well-characterized effects on translation initiation [64].

Finally, the data also indicate that the degradation of defective 18S rRNA does not require the Rqt complex and differs from NRD, as neither Mag2 nor Rps3 ubiquitination are required, thus exposing differences between these decay pathways.

### Bypassing Rio1 stabilizes miscleaved 18S rRNAs

Above, we have shown that Nob1 has limited sequence specificity as it cleaves the truncated 18S rRNA mutant constructs, which present an incorrect cleavage site (Fig 2B), consistent with observations that demonstrate miscleavage in vitro [5,22–24]. Note that the degradation of mature, tagged 18S rRNA reveals the existence of 20S pre-rRNA in the strains expressing the “miscleaved” rRNAs, which is normally “hidden” under the much more abundant 18S rRNA. In addition, it is possible that the forced expression of these “miscleaved” rRNAs depletes free Nob1, or Dom34, which would lead to 18S rRNA processing defects [3]. Yet, we have also shown that the majority of 18S rRNA in wt cells is cleaved correctly and that maintaining accurate cleavage is important because miscleaved rRNAs, even in small amounts, induce collisions that perturb translation globally. Together, these observations raise the question: How do cells maintain fidelity given that it is not entrusted to Nob1? Clearly, decay of miscleaved rRNAs after maturation is part of the answer as demonstrated above. This pathway, if overloaded, induces a cellular stress response [83]. Moreover, miscleaved 18S rRNAs are dominant negative (Fig 3A) even if they make up only approximately 5% of total cellular 18S rRNA [44,88,89]. Thus, reducing the amount of miscleaved 18S rRNA that enters the translating pool is critical.

We therefore hypothesized that correct 18S rRNA cleavage was monitored by the Rio1-mediated checkpoint that prevents release of immature rRNA into the translating pool [7]. This checkpoint is established as Nob1 and Pno1 cooperate to prevent pre-40S ribosomes from initiating translation prematurely. Nob1 and Pno1 are released by the kinase Rio1, but only after Nob1 has cleaved 18S rRNA [7,30]. Thus, this QC checkpoint ensures that only mature, 18S rRNA-containing ribosomes engage in translation [7]. Importantly, if Rio1 required cleavage accuracy, this checkpoint could also allow for monitoring of correct cleavage of the 3′-end of 18S rRNA. If this hypothesis is correct, we would expect miscleaved 18S rRNAs to be more abundant in cells that bypass this QC step.

**Fig 6:**
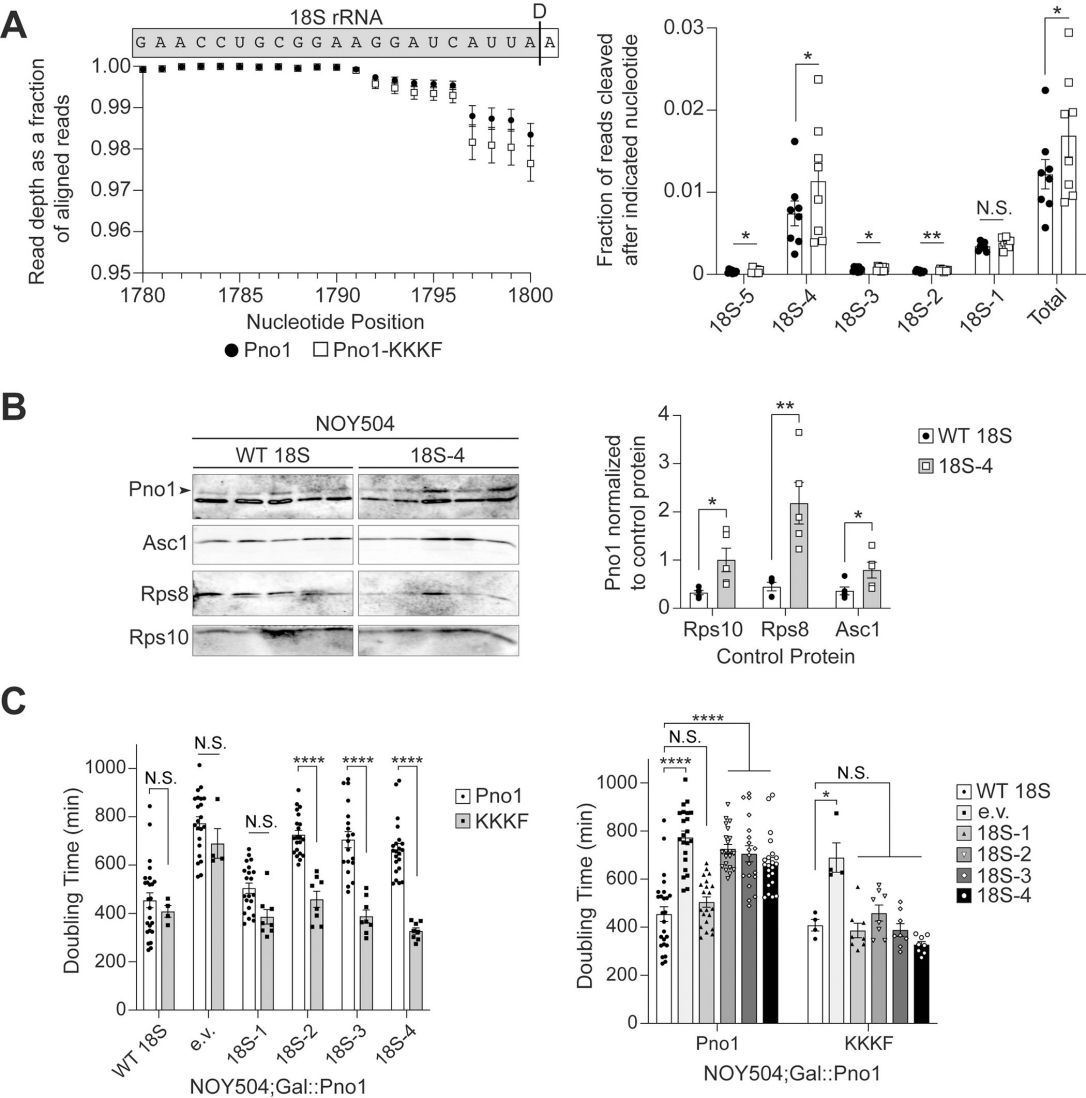
Bypassing Rio1 stabilizes miscleaved 18S rRNAs. (A) 3′-RACE-sequencing of 18S rRNAs extracted from 40S ribosomal subunits purified from Gal::Pno1; Gal::Dim1 cells depleted of endogenous Pno1 and Dim1 by growth in glucose and supplemented with plasmids expressing Dim1-E85A and either Pno1 or Pno1-KKKF (K208E/K211E/K213E/F214A), which bypasses the Rio1-mediated QC step during pre-40S ribosome assembly [7]. The WT Pno1 data is the same as in Fig 1A and 1B. Left: Read depth at each nucleotide normalized to the number of reads aligned to the 3′-end of 18S rRNA, upstream of the cleavage site. Above the graph is a schematic of the 18S rRNA and the ITS1 sequence above their corresponding nucleotide position and read depth. A black line indicates the D cleavage site that forms the 3′-end of 18S rRNA. Right: The fraction of reads miscleaved after each of the final 5 nucleotides of 18S rRNA. “Total” represents the cumulative miscleavage from 18S-5 to 18S-1. Data are the average of 8 biological replicates, and error bars indicate SEM (error bars are too small to be seen for many data points). N.S. not statistically significant, *p < 0.05, **p < 0.01, by ratio paired *t* test comparing miscleavage in Pno1 and Pno1-KKKF for each nucleotide. Pno1 and Pno1-KKKF samples grown and analyzed on the same day were considered paired replicates. (B) Left: Western blot of MS2-tagged RNA affinity-purified ribosomes containing WT or miscleaved 18S-4 rRNA. Both contain an internal MS2 RNA hairpin and are transcribed from a plasmid (GPD promoter). NOY504 cells were grown at 37°C. The arrowhead notes the upper band corresponding to Pno1. Right: Quantification of Pno1-bound ribosomes normalized to either Rps10, Rps8, or Asc1 control proteins as indicated. Data are the average of 5 biological replicates, and error bars indicate SEM. *p < 0.05, **p < 0.01, by unpaired *t* test comparing Pno1 abundance on WT 18S to 18S-4 ribosomes for each control protein. (C) Doubling times of NOY504;Gal::Pno1 cells grown at 37°C, depleted of endogenous Pno1 by growth in glucose, and expressing either Pno1 or Pno1-KKKF and either WT 18S, an empty vector (e.v.), or miscleaved 18S rRNAs from a GPD promoter. Data are the averages of 4–25 biological replicates, and error bars indicate SEM. N.S. not statistically significant, *p_adj_ < 0.05, ****p_adj_ < 0.0001, by two-way ANOVA (Tukey’s multiple comparisons test). The graph on the right is the same data as the graph on the left, presented in a different order for clarity in representing statistical comparisons. Raw numerical values to make panels A, B, and C are available as Supporting Information under **S6 Data**. QC, quality control; RQC, ribosome-associated quality control; WT, wild type.

To test this prediction, we first introduced a mutation in Pno1 that bypasses this QC step. Pno1-KKKF (K208E/K211E/K213E/F214A) disrupts Pno1’s contact at the 3′-end of 18S rRNA (S7A Fig) thereby weakening the binding of Pno1 to the pre-40S ribosome [90], resulting in Rio1-independent release of Pno1 (and Nob1 which is weakly bound in the absence of Pno1 [7]). We performed 3′-RACE-sequencing of 18S rRNA from 40S ribosomes purified from cells depleted of endogenous Pno1 and Dim1 and expressing wt Pno1 or Pno1-KKKF and the dimethylation-deficient Dim1-E85A [69]. Approximately 96% to 99% of the 2.8 to 4.5 million reads per sample we obtained map to the 3′-end of 18S rRNA, and 2.5% of stable 18S rRNAs are miscleaved in cells expressing Pno1-KKKF, compared to only 1.9% miscleavage in WT cells (Figs 6A and S7B). Specifically, products miscleaved upstream of the correct 18S rRNA 3′-end are more abundant in cells expressing Pno1-KKKF compared to cells expressing wt Pno1 (Figs 6A and S5C). In agreement with no growth defect caused by 18S-1 (Figs 2A and 3A), miscleavage at 18S-1 was not significantly different in cells expressing wt Pno1 or Pno1-KKKF. Miscleavage in ITS1 downstream of the 18S rRNA 3′-end is also not significantly altered upon Pno1-KKKF expression (S6B and S6C Fig). Importantly, the distribution of miscleavage events is the same in wt Pno1 and Pno1-KKKF cells (Fig 6A), demonstrating that changes in the overall rate of miscleavage were not due to changes in cleavage site recognition by Nob1 and indicating that Nob1 activity remained unperturbed. Thus, bypassing Rio1 increases the abundance of miscleaved 18S rRNAs. Importantly, this is specific to 18S rRNA 3′-end cleavage, as bypassing Rio1 does not affect miscleavage at the 3′-end of 25S rRNA (S6D Fig).

If the Rio1-mediated checkpoint is involved in surveillance of 18S rRNA miscleavage, as suggested by the data above, then we would expect that miscleaved 18S rRNA that escape into the translating pool retain Pno1. To test this prediction and further confirm a role for Rio1 in monitoring cleavage accuracy, we grew NOY504 cells at 37°C expressing either wt 18S rRNA or miscleaved 18S-4 rRNA containing MS2 hairpins loops and purified plasmid-derived ribosomes via MS2-tagged RNA affinity purification. Indeed, western blots indicated that relative to 3 RPs, Pno1 is significantly more abundant on the ribosomes containing −4 miscleaved 18S rRNA than their wt counterparts (Fig 6B). Unfortunately, we were unable to measure whether Nob1 is also retained on miscleaved ribosomes because the bait protein for purification, MS2-MBP, co-migrates with Nob1 on a western blot, and cross-reacts with the Nob1 antibody at the concentrations of the experiment.

To further validate that miscleaved 18S rRNA-containing ribosomes retain Pno1, we tested whether weakly binding Pno1 mutants could partially rescue the slow growth phenotype of miscleaved 18S rRNAs. Pno1-KKKF rescues the growth defect of cells expressing miscleaved 18S rRNAs truncated by 2 to 4 nucleotides, with no significant effect on the growth of cells expressing miscleaved 18S rRNAs truncated by a single nucleotide (Fig 6C). Therefore, part of the growth defect arises because Pno1 cannot be removed from miscleaved ribosomes, showing that miscleaved ribosomes fail the QC checkpoint mediated by Rio1.

### Rio1 binds miscleaved RNAs more weakly

We have previously observed that overexpression of Rio1 in the presence of an inactive Nob1 mutant releases 20S pre-rRNA-containing ribosomes into the translating pool [7], indicating that Rio1’s selectivity arises from differences in its affinity for 20S or 18S-containing ribosomes. We therefore wanted to test directly whether Rio1 was able to distinguish correctly from incorrectly cleaved rRNAs based on differential binding affinity. To test this hypothesis, we used a previously described quantitative in vitro RNA-binding assay [21] to measure the binding of Rio1 to in vitro transcribed RNA mimics of 18S rRNAs with variable 3′-ends. These mimics contained the 3′-end of 18S rRNA, starting at h44, the penultimate helix in 18S rRNA, and ending at either the correct 3′-end, 3 nucleotides further, or 4 nucleotides short. The RNAs were transcribed from PCR products generated with primers containing two 2′-O-methylated RNA nucleotides to ensure the precision of the 3′-end [91], folded, and then incubated with Rio1. Rio1-bound and free RNAs were separated by native PAGE gels. Comparing the binding of Rio1 to these 18S rRNA mimics, our data show 3-fold stronger Rio1 binding to the RNA mimic of the correctly cleaved 18S rRNA (H44-D) compared to the RNA mimic of the truncated, miscleaved 18S rRNA (H44-D-4). Rio1 also binds the correctly cleaved 18S rRNA slightly (1.4-fold) stronger than its binding to the elongated, miscleaved 18S rRNA (H44-D+3, Fig 5A and 5B). The preferential binding to accurately processed 18S rRNA suggests that Rio1 directly senses the sequence and/or length at the 3′-end of 18S rRNA.

**Fig 7:**
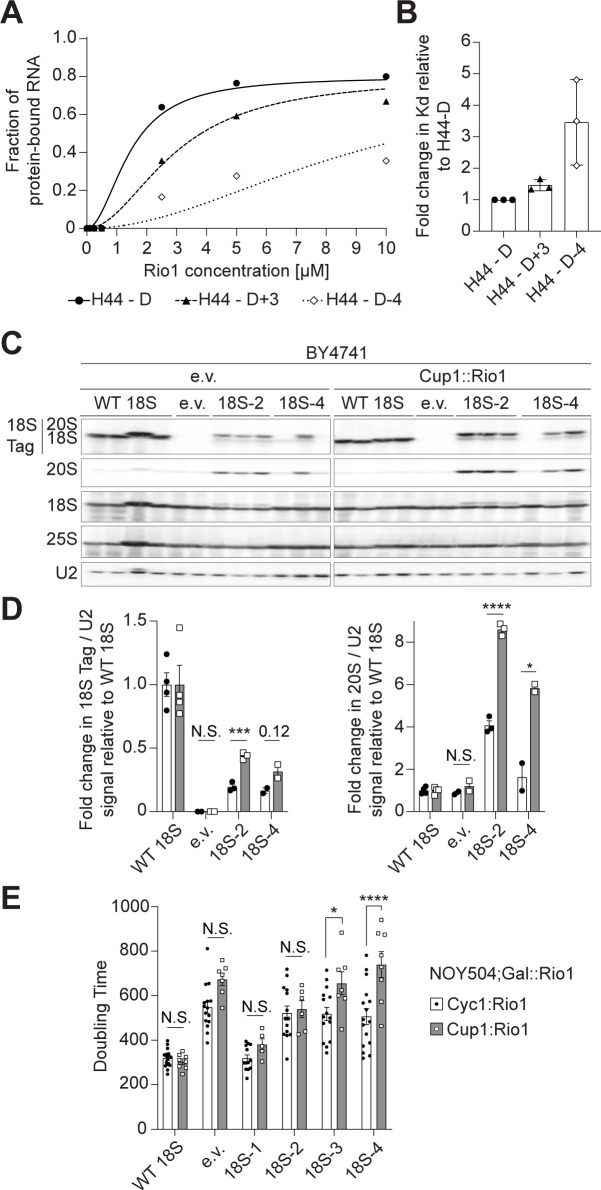
Rio1 monitors 18S rRNA cleavage during pre-40S assembly QC. (A) Representative RNA-binding assay with in vitro transcribed H44-D (18S rRNA mimic, black circles), H44-D+3 (+3 nt, long miscleaved 18S rRNA mimic, black triangles), and H44-D-4 (−4 nt, short miscleaved 18S rRNA mimic, white diamonds) RNAs and recombinant Rio1. Three independent experiments resulted in K_*d*_ = 2.3 +/− 1.0 μm^2^ for Rio1 binding H44-D, K_*d*_ = 3.3 +/− 1.2 μm^2^ for Rio1 binding H44-D+3, K_*d*_ = 7.5 +/− 1.9 μm^2^ for Rio1 binding H44-D-4. (B) To account for day-to-day variations, K_*d*_ values of Rio1 binding each RNA mimic from panel B were normalized to the K_*d*_ for Rio1 binding H44-D on each gel (fold change = 1). (C) Total RNA northern blot from BY4741 cells expressing excess Rio1 under a Cup1 promoter or only endogenous Rio1 (e.v., empty vector) and either WT 18S, an empty vector, or miscleaved 18S rRNAs from a Gal7 promoter. (D) Quantification of northern blots in panel C. Plasmid-encoded 18S rRNA or total 20S pre-rRNA was normalized to U2 snRNA. 18S Tag/U2 ratios from cells expressing miscleaved 18S rRNAs were normalized to the 18S Tag/U2 ratios from cells expressing WT 18S for each cell background (fold change = 1). Data are the averages of 2–4 biological replicates, and error bars indicate SEM. N.S. not statistically significant, *p < 0.05, ***p < 0.001, ****p < 0.0001, by unpaired *t* test for each 18S rRNA variant. (E) Changes in doubling time of NOY504;Gal::Rio1 cells grown at 37°C, depleted of endogenous Rio1 by growth in glucose, expressing Rio1 either under a Cyc1 promoter (near endogenous expression level) or a Cup1 promoter (high expression level) and either WT 18S, an empty vector, or miscleaved 18S rRNAs from a GPD promoter. Cells expressing Cup1::Rio1 were grown in media supplemented with 10 μm CuSO_4_ to activate the Cup1 promoter. Data are the averages of 5–17 biological replicates, and error bars indicate SEM. N.S. not statistically significant, *p < 0.05, ****p < 0.0001, by unpaired *t* test for each 18S rRNA variant. Raw numerical values to make panels B, D, and E are available as Supporting information under **S7 Data**. QC, quality control; rRNA, ribosomal RNA; WT, wild type.

To further test if weakened binding to miscleaved RNAs in vivo could account for Rio1’s ability to QC cleavage accuracy, we tested whether we could rescue Rio1’s weakened binding to miscleaved 18S rRNA-containing ribosomes by overexpressing Rio1. If so, then we expect that miscleaved 18S rRNAs should become more abundant. Indeed, in cells expressing endogenous rRNA (BY4741) and excess Rio1 (Cup1 promoter, S8 Fig), we observed increased accumulation of miscleaved 18S rRNA (Fig 7C and 7D). We also saw an increase in 20S pre-rRNA abundance, as the extra Rio1 also releases Nob1 and Pno1 prematurely from un-cleaved ribosomes [7]. Next, we grew NOY504 cells at 37°C to express only plasmid-encoded 18S rRNAs and depleted endogenous Rio1 under a galactose-inducible/glucose-repressible promoter by growth in glucose. Rio1 was then expressed from a plasmid, either near endogenous levels under the Cyc1 promoter or overexpressed under the copper inducible Cup1 promoter (S7 Fig). As expected from an increase in miscleaved 18S rRNA-containing ribosomes, excess Rio1 caused a significant additional growth defect in cells relying solely on ribosomes containing miscleaved 18S rRNAs (Fig 7E). Altogether, these data support a model in which Rio1 monitors the precise cleavage of the 18S rRNA 3′-end during ribosome maturation, marking correctly processed ribosomes by the removal of Nob1 and Pno1 from the ribosome.

## Discussion

### Rio1 monitors 18S rRNA cleavage accuracy during ribosome assembly

**Fig 8:**
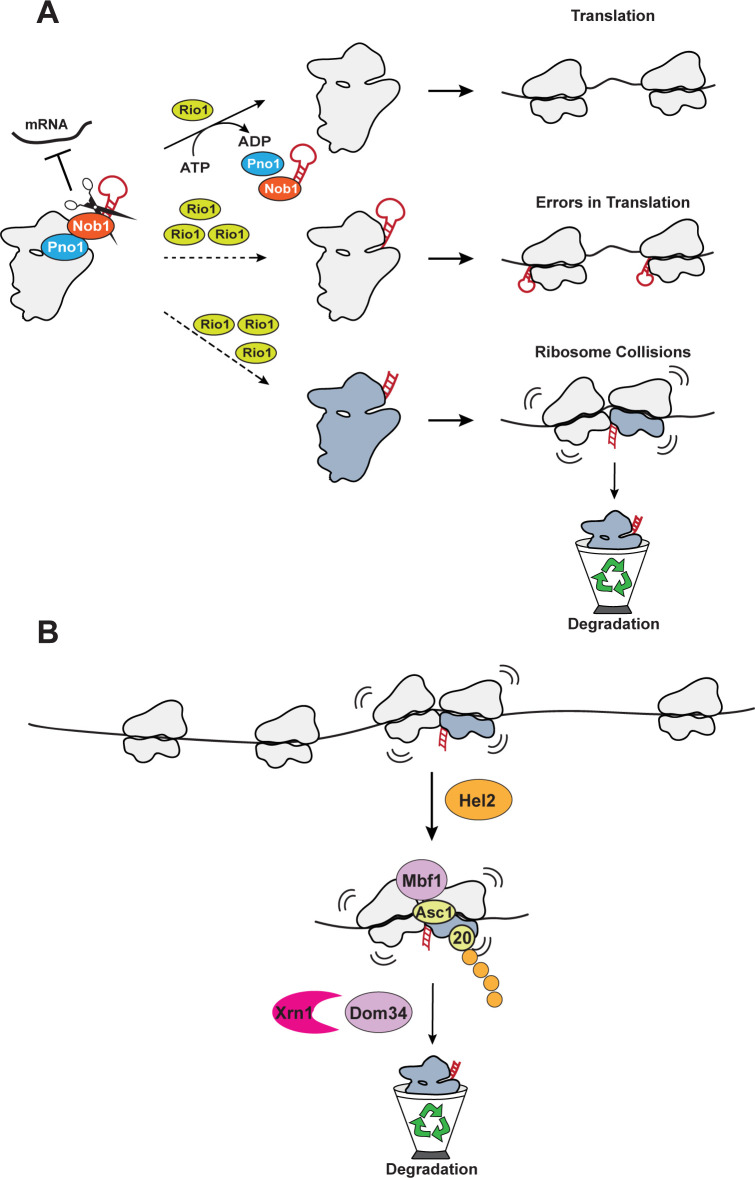
Model for quality control of miscleavage during and after assembly. (A) Rio1-mediated QC of 18S rRNA end formation. Nob1 and Pno1 establish a QC checkpoint in which Nob1 prevents mRNA recruitment to immature ribosomes containing 20S pre-rRNA. (Top) Once Nob1 cleaves the 18S rRNA to form the correct 3′-end, Rio1 releases Nob1 and Pno1 from the nascent ribosome, which can now recruit mRNA [7]. Disrupting the Rio1-mediated QC checkpoint by overexpression of Rio1 (shown here) or mutations in Pno1 [7] that weaken Pno1 binding leads to the release of immature ribosomes, which produce translational errors [7] (middle), or ribosomes containing miscleaved 18S rRNA (in blue, bottom), which lead to collisions with correctly matured ribosomes. (B) Ribosome collisions mediate the recognition of dysfunctional ribosomes. 40S ribosomes containing miscleaved 18S rRNA (shown in blue) translate more slowly, thereby leading the trailing ribosome to collide with them. The disomes are stabilized by Asc1 at the interface, bound by Mbf1, and recognized by Hel2, which ubiquitinates (orange circles) Rps20. These proteins and Dom34 and Xrn1 are required for the degradation of the miscleaved ribosome. QC, quality control; rRNA, ribosomal RNA.

In this work, we expand our understanding of the role Rio1 plays in ribosome assembly and show that Rio1 helps ensure that 18S rRNA is cleaved at the correct site. Previously, we demonstrated that Nob1 and Pno1 establish a quality control checkpoint wherein Nob1 prevents immature ribosomes containing 20S pre-rRNA from recruiting mRNA. After Nob1 cleaves 18S rRNA, Rio1 removes Nob1 and Pno1 in an ATP-dependent manner from nascent ribosomes, thus licensing only mature ribosomes to recruit and translate mRNA ([7], Fig 8A, top).

In the current work, we show that Nob1 can miscleave its substrate in vivo, confirming earlier observations that purified recombinant Nob1 miscleaves RNA in vitro [5,22–24]. Miscleaved rRNAs are scarce in vivo but become more abundant upon bypass of the Rio1-mediated QC step (Figs 1 and 6). Thus, our data support a model in which Rio1 monitors correct cleavage, restricting miscleaved ribosomes from entering the translating pool. In vitro RNA binding data show that Rio1 binds correctly cleaved RNA more strongly than miscleaved RNA (Fig 7A and 7B) and overexpression of Rio1 increases the amount of miscleaved RNA in vivo (Fig 7C and 7D). Thus Rio1’s ability to monitor correct rRNA cleavage reflects differences in rRNA binding affinity. The importance of this QC step is underlined by our observation that miscleaved rRNAs, even in small quantities, disturb translation.

### Miscleaved 18S rRNAs are more frequently truncated than elongated

Through our sequencing analysis, we discovered that in vivo, miscleaved 18S rRNAs were truncated within 18S rRNA 10-fold more frequently than they were elongated and cleaved within ITS1 (Fig 1). This is different from in vitro observations, where Nob1 equally miscleaves 3′ and 5′ of the canonical 18S rRNA cleavage site [5,22–24]. To reconcile these observations, we consider the differences of Nob1-mediated rRNA cleavage in vitro and in vivo. In vitro, radiolabeling of RNAs allows detection of the elongated and truncated 18S rRNAs. Meanwhile, in vivo, if Nob1 miscleaves 18S rRNA 3′ to its cleavage site, the elongated 18S rRNA retains Nob1, preventing these miscleaved ribosomes from entering the translating pool [7]. Nob1 then has a second chance to cleave at the correct nucleotide, thereby correcting its previous mistake. However, if Nob1 truncates 18S rRNA by miscleaving 5′ to its cleavage site within mature 18S rRNA, the Nob1 binding site on the rRNA will be removed. Nob1 would then dissociate from the ribosome, releasing the truncated, miscleaved rRNA-containing ribosomes into the translating pool, where they can be detected in our sequencing assay, and from where they are also cleared by ribosome collisions. Thus, we postulate that the differential abundance of the elongated and shortened miscleavage products reflect Nob1 being able to cleave elongated 18S rRNAs again, rather than the ability of Nob1 to effectively discriminate against 3′-elongated sequences.

Intriguingly, Nob1 appears to prefer cleaving its rRNA substrate 5′ to an adenosine, as the most abundant miscleavage sites in vivo are all followed by an adenosine (Fig 1). Moreover, miscleavage of rRNA substrates by Nob1 in vitro also occurs upstream of an adenosine [5,22,24]. Furthermore, while Nob1 is conserved in most archaea, the sequence at the 3′-end of the small subunit rRNA is not. Even so, in the archaea *Pyrococcus horikoshii*, Nob1 cleaves 5′ to an adenosine to form the canonical 16S rRNA 3′-end [24]. While base-specific interaction between Nob1 and pre-18S rRNA cannot explain this (very limited) sequence specificity (S9 Fig, [30]), we note that the 2′-OH of the +1A is within hydrogen bonding distance to both the carbonyl oxygen as well as the side-chain hydroxyl of threonine 237 (human numbering), a residue conserved from archaea to humans. This might align the rRNA, which adopts multiple positions in the structures of assembly intermediates at different stages [30,92]. Notably, when overlaying these distinct rRNA positions onto Nob1 in its active position, either the correct cleavage site, or the -1 miscleavage site are in the Nob1 active site, indicating that a shifting rRNA position contributes to the lack of cleavage specificity.

### Monitoring rRNA cleavage

Our data demonstrate the importance of forming the canonical 3′-end of 18S rRNA. The 3′-end of 18S rRNA is highly conserved from yeast to humans (S2A Fig) and our data show that miscleaved 18S rRNAs provide a 1.4- to 1.8-fold growth defect (Fig 2A). While Nob1 can miscleave its rRNA substrate in vitro and in vivo, we observe very little miscleaved rRNA in cells with functional QC mechanisms. While we cannot rule out that Pno1 remaining on these pre-40S ribosomes contributes to the growth defect, as it blocks binding of the essential RP Rps26 [30,32–34], the observation that excess Rio1 removes Nob1 and Pno1 from otherwise stalled substrates [7], but exacerbates the growth defects (Fig 7E) argues against this. Either way, Pno1 bound to miscleaved ribosomes (Fig 6B) is a consequence of the failed QC step in which Rio1 does not remove Nob1 or Pno1 from miscleaved ribosomes. This supports our conclusion that cleavage accuracy at the 3′-end of 18S rRNA is quality controlled, and bypass of this QC step leads to dysfunctional ribosomes in the translating pool, where they disrupt translation by correctly processed ribosomes (Fig 8).

### Rio1 concentration is critical for proofreading of 18S rRNA cleavage

Our current work along with previous observations indicates that the concentration of Rio1 is critical for monitoring correct rRNA cleavage and maintaining this QC step. Rio1 has a lower binding affinity for miscleaved rRNAs than correctly cleaved rRNAs in vitro (Fig 7A and 7B). Thus, increasing the amount of available Rio1 leads to Rio1 binding to non-optimal substrates, releasing immature and miscleaved ribosomes into the translating pool (Fig 7C and 7D). Therefore, the stringency of this QC step, the gate separating late-stage immature pre-40S and mature 40S ribosomes in the cytoplasm, relies on the concentration of Rio1 relative to that of the nascent ribosome pool. Interestingly, whole-genome sequencing of cancer cells revealed that human Rio1, RIOK1, is frequently amplified in cancer: 7% of ovarian tumors, 3.6% of melanomas, 4.2% of mature B cell neoplasms, 3.8% of ocular melanomas, 3.4% of bladder urothelial carcinomas, 2.7% of liver hepatocellular carcinomas, 2.8% of cholangiocarcinomas, and 2.3% of mesotheliomas (The Cancer Genome Atlas (TCGA): https://www.cancer.gov/tcga, https://www.cbioportal.org). Moreover, RNAseq data show that relative to the mRNAs encoding for RPs, which are a measure of flux though the ribosome assembly pathway, most cancer cells display increased abundance of RIOK1, and RIOK1 abundance is increased more than the close relative RIOK2, or hCINAP, the human homolog for the ATPase Fap7, which both function directly prior to Rio1 [3,7,93] (S9 Fig). While it remains unknown whether these changes in RIOK1 abundance play any role in promoting cancer progression by releasing miscleaved 18S rRNA-containing ribosomes into the translating pool, moving forward it will be important to understand how fluctuations in the relative concentrations of assembly factors affect QC during ribosome assembly and translation in human cells.

### Miscleaved ribosomes translate more slowly

Ribosome runoff experiments indicate that ribosomes containing miscleaved 18S rRNA translate more slowly, consistent with their accumulation in polysomes, when they are the only ribosomes. While we do not know the origin of this translation defect, it seems unlikely that it arises from a direct effect on the rate of peptide bond formation as peptides are formed on the large subunit. Given that the 18S rRNA 3′-end is located on the platform, which comprises the E-site, we speculate that impaired E-site tRNA binding could be responsible for this defect, e.g., defective E-site tRNA binding might slow translocation by destabilizing transitions to the later translocation stages where the E-site is filled.

### Degradation of defective ribosomes

Ribosomes stalled on defective mRNAs lead to collisions with subsequent ribosomes [37–41]. The unique Asc1-containing interface between these collided disomes is then recognized by a large machinery, including Hel2, which ubiquitinylates Rps20, ultimately leading to the degradation of the defective mRNA as well as the disassembly of the collided disomes, either via the Rqt complex (RQC) or via Dom34 (NGD, S1B Fig). Nonfunctional 18S rRNA decay (NRD, S1A Fig) targets for decay 18S rRNAs containing a mutation in its decoding center (18S:A1492C), stalled at the start codon due to its inability to bind an incoming tRNA. 18S NRD also requires Asc1 as well Rps3 ubiquitinylation by Mag2. Whether this involves a collision event (presumably with scanning 40S) remains unclear.

Here, we show that in the presence of mature ribosomes, functionally compromised miscleaved rRNAs are rapidly degraded in a collision-dependent pathway (Fig 8B). Our data show that ribosomes containing miscleaved rRNAs translate more slowly, which in the presence of correctly cleaved ribosomes leads to collisions by subsequent ribosomes. As in RQC and NGD, the collided disomes are stabilized by Asc1 and recognized by Mbf1 and the E3 ligase Hel2, which ubiquitinates Rps20. How the defective ribosomes in the collided disomes are decayed, and whether bound mRNAs are also degraded, remains to be studied further: Data in here indicate that the decay of miscleaved ribosomes does not require the Rqt complex but appears to utilize the NGD factors Dom34 and Cue2, although the role of Cue2 appears complicated and is not well-defined by the work in here. This observation could either indicate that disassembly of ribosome collisions arising from miscleaved defective ribosomes is fundamentally different from collisions arising from defects in the mRNA, perhaps reflecting the fact that these collided disomes might translate together, instead of being stalled on mRNA. Alternatively, it is possible that disassembly of the collided ribosomes is not required for their degradation. Regardless, in recognition of the differences of this newly discovered pathway to RQC, NGD, and NRD, we suggest naming it dysfunctional rRNA decay (DRD).

### Implications for ribosome heterogeneity

Herein, we show that ribosomes with altered function, even at low concentrations, perturb the translation of correctly matured ribosomes, inviting collisions with trailing ribosomes, and ultimately leading to the degradation of the ribosomes with altered function. In the case herein, the ribosomes with altered function contain miscleaved 18S rRNA, but the same should be true for ribosomes lacking modifications in the rRNA or RPs, or missing RPs that affect the ability of the ribosome to bind A- or P-site tRNA or to translocate efficiently, thereby slowing down elongation. The possibility of heterogeneous ribosomes arising from differences in rRNA modifications in functionally important regions has been raised and it has been speculated that they could affect global gene regulation [94–96]. The data here indicate that this is unlikely to be the case because such heterogeneous populations, if they existed, would be rapidly homogenized via DRD, dysfunctional RNA decay. Nonetheless, ribosomes of altered function could persist if they sort themselves onto distinct classes of mRNAs, as we have shown for ribosomes containing and lacking Rps26 [18], or if they occur in cells with low translational load, including neurons [97].

## Materials and methods

### Yeast strains and cloning

*Saccharomyces cerevisiae* strains used in this study were obtained from Euroscarf, the Yeast Knockout Collection from GE Dharmacon (now Horizon Discovery Biosciences), or were made using PCR-based recombination [98]. Strain identity was confirmed by PCR, quantitative growth assays, and western blotting when antibodies were available. Site-directed mutagenesis was used to create mutations in plasmids, which were confirmed by sequencing. Plasmids were propagated in XL1 Blue competent cells. Yeast strains and plasmids used in this study are listed in S1 and S2 Tables, respectively.

### rRNA 3′-RACE sequencing

#### Ribosome purification

Gal::Pno1; Gal::Dim1 cells supplemented with plasmids encoding Dim1-E85A and either Pno1 or Pno1-KKKF were depleted of endogenous Pno1 and Dim1 by growth in YPD for 8 doublings at 25°C or 30°C. Cells were harvested between OD 1.0 and 2.0. Ribosomes were purified as previously described [99]. Ribosomal subunits were separated during purification and stored at −80°C as individual subunits. Due to concerns that reverse transcription through the Dim1-dimethylation site in 18S rRNA (m^6^_2_A1781 and m^6^_2_A1782) would pose complications, we used the methylation-incompetent Dim1-E85A mutation [69] to allow us to sequence the final 40 to 60 nucleotides of 18S rRNA.

#### Library preparation and Illumina sequencing

18S and 25S rRNA was isolated from 40S and 60S ribosomal subunits, respectively, by phenol-chloroform-isoamyl alcohol extraction. rRNA was treated with Dnase I (New England Biolabs (NEB)) and size selected on a denaturing TBE-Urea-PAGE gel. rRNA was extracted from the gel by freeze-thawing the gel pieces in RNA extraction buffer (300 mM NaOAc, 1 mM EDTA, and 0.25% SDS) and ethanol precipitated. rRNA 3′-ends were dephosphorylated using rSAP (NEB) and ligated to either the Universal miRNA Cloning Linker (NEB; 25S rRNA samples and some 18S rRNA samples) or ligated to a pre-adenylated linker containing a UMI (unique molecular identifier, Integrated DNA Technologies (IDT), some 18S rRNA samples) using truncated T4 RNA ligase K227Q (NEB) to protect the 3′-end of the rRNAs from degradation and to identify the true rRNA 3′-end after sequencing (S3 Table). Linkers were added in 2-fold excess of rRNA ends, and 5′-ends of the DNA linkers and the rRNA were deadenylated by 5′-deadenylase and the excess linkers were degraded by DNA-specific RecJ_f_ exonuclease (NEB). rRNA was reverse transcribed using a linker-specific primer and Protoscript II RT (NEB) to generate the first strand of cDNA. After Rnase H (NEB) treatment, the second strand was synthesized using an 18S or 25S rRNA-specific forward primer, a linker-specific reverse primer, and Q5 high-fidelity DNA polymerase (NEB). The 18S rRNA-specific primer was designed to sequence either the last 41 or 62 nt of 18S rRNA, while the 25S rRNA-specific primer was designed to sequence the last 47 nt of 25S rRNA. The forward and reverse second strand synthesis primers contained partial P5 and P7 Illumina sequencing adapters, as indicated in S3 Table. The cDNA was purified either on a denaturing TBE-Urea-PAGE gel and extracted by freeze-thawing in DNA extraction buffer (300 mM NaCl, 10 mM Tris-HCl (pH 8.0), and 1 mM EDTA) or cleaned up with AMPure XP beads (Beckman Coulter). The purified cDNA was amplified via PCR to generate the final libraries and add the complete P5 and P7 Illumina adapter sequences. The final size of the 25S cDNA libraries was about 194 bp, with a 64 bp insert, whereas the 18S cDNA libraries were either about 188 bp with 58 bp insert, or about 251 bp with 121 bp insert. Shorter 18S cDNA libraries were generated using the Universal miRNA Cloning Linker and the longer 18S cDNA libraries were generated using the UMI-containing linker. Library size and quality were assessed on an Agilent 2100 Bioanalyzer (Agilent Technologies). Validated libraries were pooled at equimolar ratios and loaded onto the NextSeq 500 flow cell. Primers for the reverse transcription and second strand synthesis reactions are listed in S3 Table. All enzymes were bought from NEB and were used according to the manufacturer’s recommendations.

#### Bioinformatic processing

Demultiplexed and quality-filtered raw reads (fastq) generated from the NextSeq 500 were trimmed to remove Illumina adapter sequences with Trim Galore! (version 0.6.1, Babraham Bioinformatics). Only reads containing the full linker sequence were retained, and the linker sequences were removed with CutAdapt (version 3.5, [100]) using Python version 3.8.3. Quality of trimmed reads was assessed using FastQC (version 0.11.4, Babraham Bioinformatics). Trimmed reads were aligned to the *S*. *cerevisiae* 35S rDNA sequence (S288C) from the Saccharomyces Genome Database [101] with Bowtie2 (version 2.2.9, [102]). SAMtools (version 1.1.0, [103,104]), BAMtools (version 2.4.0, [105]), and BEDtools (version 2.17.0, [106]) were used to identify reads aligning to the reference genome and to calculate the read depth or the fraction of reads cleaved at each nucleotide position. For 18S cDNA samples containing a UMI, the program UMI-tools (version 1.1.2, [107]) was used to extract the UMI sequence from each read prior to alignment. The extracted UMI sequence was then used to deduplicate reads after alignment but prior to calculating read depth and cleavage.

#### Quantitative growth assay

NOY504 cells expressing plasmid-derived 18S rRNAs were grown overnight at 30°C in glucose dropout media, diluted for a day culture in the same media, and grown for 3 h at 30°C followed by 37°C for 5 h until the cells reached mid-log phase. The cells were then inoculated into a 96-well plate in YPD media at OD 0.1. Cells expressing plasmid-derived 18S rRNAs with a BY4741 background (as indicated in S1 Table) were grown overnight, diluted, and grown for an additional 3 h to mid-log phase in glucose dropout media at 30°C. These cells were then inoculated into a 96-well plate in YPD media at OD 0.05. Cells expressing Pno1 or Pno1 mutants with and without Rio1 were prepared as previously described [7]. Cells were grown at 30°C or 37°C, as indicated in the figure legend, while shaking and the doubling times were measured in a Synergy 2 multimode microplate reader (BioTek).

### Sucrose density gradient analysis

Sucrose density gradient analysis and polysome profiling of whole-cell lysates followed by western blotting and Northern blotting were performed as previously described [3,7]. All cells were grown to mid-log phase and then harvested. NOY504 cells expressing plasmid-derived 18S rRNAs were depleted of their endogenous ribosomes by growth at 37°C for 7 cell doublings prior to harvesting. Essential, endogenous proteins expressed under a Gal1 promoter were depleted in cells by growth in glucose dropout media for at least 12 h prior to harvesting. The percent of 18S rRNA or 20S pre-rRNA in the polysome fractions was calculated by dividing the amount of 18S rRNA or 20S pre-rRNA in the polysome fractions (fractions 8–13) by the total amount of 18S rRNA or 20S pre-rRNA in all fractions (fractions 2–13). The percent of free Pno1 was calculated by dividing the amount of non-ribosome bound (free) Pno1 (fractions 1–2) by the total amount of Pno1 in all fractions (fractions 1–13).

### Ribosome run-off assays

NOY cells were grown at 34°C for at least 4 doublings to an OD of 1. LTM was added for a final concentration of 2.9 μm LTM and three 50 ml samples were collected by rapid filtration approximately 1, 2, and 5 min after addition of LTM. In addition, a control sample without LTM addition was also collected. Samples were ground together with frozen pellets of lysis buffer in liquid N_2_. Lysates were spun on 10% to 50% sucrose gradients and fractionated as described above. 80S ribosomes and polysomes were quantified via the area under the A254 nm absorption curve.

### Northern analysis

Northern blotting was carried out essentially as previously described [3] using probes listed in S3 Table. For whole-cell RNA northern blots, cells were grown in glucose dropout media at 30°C, except NOY504 cells which were grown at 37°C, and 10 ml of cells at OD 0.5 were harvested, total RNA was extracted, and 5 μg of RNA per sample was used for the northern blots.

### Pulse-chase measurement of rRNA decay

BY4741 cells transformed with galactose-inducible wt or mutant rRNA plasmids were grown overnight in galactose dropout media to an OD of 0.3. A 15-ml sample of cells from each culture was collected before spinning down the cells, resuspending them in YPD, and continuing to incubate at 30°C. A total of 15 ml aliquots of cells were collected every 20 min for 2 h by centrifugation at 3,000×g for 10 min. Samples were stored at −80°C before RNA extraction and northern blot analysis.

### Protein expression and purification

Rio1 was purified as previously described [7]. Expression and purification of the maltose-binding protein (MBP)-MS2 fusion protein was performed as described, with minor changes [108,109]. In brief, Rosetta DE3 competent cells transformed with a plasmid encoding His-MBP-tagged MS2 were grown to mid-log phase at 37°C in LB media supplemented with 2% glucose and the appropriate antibiotics. MBP-MS2 expression was induced by addition of 1 mM IPTG (isopropyl β-D-thiogalactoside), and cells were grown for another 5 h at 30°C. Cells were lysed in 20 mM HEPES (pH 7.9), 200 mM KCl, 1 mM EDTA, 1 mM phenylmethylsulfonyl fluoride plus tablets of proteinase inhibitor mixture (Roche) by sonication on ice. The supernatant was applied to amylose resin and the MBP-MS2 protein was eluted in a buffer containing 20 mM HEPES (pH 7.9), 20 mM KCl, 1 mM EDTA, and 10 mM maltose. The protein was dialyzed into a buffer containing 20 mM HEPES (pH 7.9), 20 mM KCl, 1 mM EDTA, and 5 mM 2-Mercaptoethanol. The protein was then purified over a MonoQ column in a 20 mM to 1 M KCl gradient in 20 mM HEPES (pH 7.9), 1 mM EDTA, and 5 mM 2-Mercaptoethanol. Finally, the protein was dialyzed into a buffer containing 20 mM HEPES (pH 7.9), 100 mM KCl, and 10% glycerol. Protein concentration was determined with a Bradford assay.

### MS2-tagged RNA affinity purification (MS2-TRAP)

MBP-MS2-bound amylose resin was prepared in advance. First, 100 μl amylose resin was added to columns and washed 4 times with 1 ml H_2_O, and then 3 times with 1 ml MS2 storage buffer (20 mM HEPES (pH 7.4), 100 mM KCl, and 10% Glycerol). Purified MBP-MS2 protein (0.2 mg) was bound to amylose resin in 1 ml MS2 wash buffer (20 mM HEPES (pH 7.4), 200 mM KCl, 1 mM EDTA, and EDTA-free protease inhibitor (Roche)) by incubating at 4°C for 1 h on a nutator. The amylose resin was then washed 4 times with 1 ml MS2 wash buffer and equilibrated with 2 ml ribosome lysis buffer (20 ml HEPES (pH 7.4), 200 mM KOAc, and 2.5 mM Mg(Oac)_2_). At this point, the MBP-MS2-bound amylose resin was ready for incubation with cell lysate.

Cells were grown in glucose dropout media at 37°C for 7 doublings and harvested at mid-log phase. Cells were then suspended in 0.5 ml/g of stringent ribosome lysis buffer (20 ml HEPES (pH 7.4), 200 mM KOAc, 2.5 mM Mg(Oac)_2_, 1 mM PMSF (phenylmethylsulfonyl fluoride), 1 mM DTT (dithiothreitol), 1 mM benzamidine, 1 μg/ml Leupeptin, 1 μg/ml Pepstatin, 10 μg/ml Aprotinin, and 1 mg/ml Heparin) and flash frozen in liquid nitrogen. Frozen cell pellets were additionally lysed by grinding into powder by mortar and pestle and thawed in 1 ml/g of stringent ribosome lysis buffer. Cell lysates were cleared and incubated with MBP-MS2-bound amylose resin in columns at 4°C for 1 h on a nutator.

After ribosome binding, the resin was washed 4 times with 1 ml ribosome lysis buffer and equilibrated with 1 ml ribosome wash buffer (20 mM HEPES (pH 7.4), 100 mM KOAc, and 2.5 Mg(Oac)_2_). Finally, the MS2-bound ribosomes were eluted in 2 elution steps. The first elution was in 30 μl elution buffer (20 mM HEPES (pH 7.4), 100 mM KOAc, 2.5 Mg(Oac)_2_, and 15 mM maltose) after incubating the resin with elution buffer for 10 min. The second elution was in 200 μl elution buffer. For western blot analysis, equal volume of 2× SDS-PAGE loading dye was mixed with the first elution and denatured at 95°C for 10 min before loaded onto an SDS-PAGE gel. Western blots were probed with the indicated antibodies.

### Disome formation assay

ΔAsc1 cells were grown in glucose dropout media at 30°C for approximately 7 doublings and harvested at mid-log phase. MS2-tagged RNA affinity purification was performed according to the protocol above, with shortened cell lysate and elution incubation times of 40 min and 7 min, respectively. The first elution was loaded onto 10% to 50% sucrose density gradients and split into 14 fractions. For northern blot analysis, RNA from fractions 2–13 was extracted with phenol chloroform isoamyl alcohol and precipitated with isopropanol. Equal volumes of loading dye and sample in formamide were loaded onto denaturing agarose gels. Blots were probed with oligos for 18S Tag, 18S, and 25S.

### RNA-binding assay

RNA-binding assays were performed as previously described [21]. Briefly, ^32^P-ATP-labeled H44-D, H44- D+3, or H44- D-4 RNAs, named after the structural elements that mark their start and end points, were prepared by in vitro transcription in the presence of α-ATP. D+3 indicates that the RNA ends 3 nucleotides after the cleavage site D and D-4 indicates that the RNA ends 4 nucleotides before cleavage site D. The RNA transcription templates were PCR products containing the same promoter sequence at the 5′ end upstream of the H44 start site, and two 2′-O-methylated RNA nucleotides at the 3′-end to reduce T7 RNA polymerase’s non-templated nucleotide addition activity at the 3′-end of the RNAs, thus promoting uniformity at the 3′-ends of each RNA [91]. RNAs were then gel purified, eluted via electroelution, precipitated, and resuspended in water. RNAs were folded by heating for 20 min at 55°C in the presence of 50 mM HEPES (pH 7.5) and 10 mM MgCl_2_. Trace amounts of each radiolabeled RNA were incubated with varying concentrations of Rio1 with 1 mM AMPPNP in 50 mM HEPES (pH 7.5), 100 mM KCl, and 10 mM MgCl_2_ for 30 min at 30°C. Samples were loaded directly onto a running 6% acrylamide/THEM native gel to separate protein bound from unbound RNAs. After drying the gel, phosphorimager analysis was used to quantify the gel. Bound RNA was plotted against protein concentration and fit to Eq 1 to obtain apparent binding constants using GraphPad Prism version 8.4.3 (471) (GraphPad Software, La Jolla, California, United States of America; www.graphpad.com).


$${Fraction}_{bound}=\frac{{Fraction}_{bound,max}{\left[Rio1\right]}^{2}}{{\left[Rio1\right]}^{2}+{{[K}_{d}]}^{2}}$$ Eq 1

### Antibodies

Primary antibodies against recombinant Rio1, Fap7, Rps10, Pno1, and Tsr1 were raised in rabbits by Josman or New England Peptide and tested against purified recombinant proteins and yeast lysates. The Rps8 antibody was a gift from G. Dieci and the Asc1 antibody was a gift from A. Link. The secondary antibody was anti-rabbit IgG conjugated to HRP from Rockland Immunochemicals. Blots were visualized using a BioRad ChemiDoc Imaging System.

### Quantitative and statistical analysis

Quantification of northern and western blots was performed using ImageJ 1.53a (National Institutes of Health). Statistical analysis was performed using GraphPad Prism version 8.4.3 (471) (GraphPad Software, La Jolla, California, USA; www.graphpad.com). Statistical tests used and the number of samples (*n*) are indicated in the figure legends.

## Supporting information

S1 Fig Translation-associated quality control mechanisms. (A) 18S non-functional decay (NRD) recognizes ribosomes stalled at initiation, either because they are defective [44,45,50] (shown in red), or because translation initiation is repressed [49]. The stalled 80S complexes might invite collisions with scanning 40S as shown, but regardless are ubiquitinylated on Rps3 via the E3 ligases Mag2 and Hel2. The Rqt complex splits the stalled 80S ribosomes [50] allowing for decay of the defective 40S, which also requires Dom34 [45]. (B) mRNA quality control senses ribosome collisions that arise from ribosomes stalled on specific sequences or on damaged mRNAs (red star). The collided disomes form a unique interface, which relies on Asc1, are bound by Mbf1, and are recognized by Hel2, which ubiquitinylates Rps20. In the major pathway (left), the stalled ribosomes are split by the Rqt complex, while in a minor pathway, termed no-go-decay (NGD), the endonuclease Cue2 cleaves mRNA between the 2 ribosomes, allowing the trailing ribosome to be rescued by Dom34. In both pathways, mRNA is degraded by Xrn1.
(EPS)

S2 Fig 3′-end of 18S rRNA is highly conserved from yeast to human. (A) Multiple sequence alignment of the entire 18S rDNA coding sequence from 8 species using Clustal Omega [110]. Only the last 45 nucleotides of the 18S rDNA sequences are shown. *Fully conserved residues. All sequences were obtained on December 2, 2021 from the following sources: *S*. *cerevisiae*–Saccharomyces Genome Database (Gene: RDN18-1); *S*. *Pombe–*PomBase Database, Gene: SPRRNA.43, [111,112]; *D*. *melanogaster*–National Center for Biotechnology Information (NCBI) (NCBI Reference Sequence: NR_133559.1); *X*. *laevis*–NCBI (GenBank: X02995.1); *R*. *norvegicus*–NCBI (GenBank: V01270.1); *M*. *musculus*–Ensembl genome database (Genome assembly: GRCm39); *P*. *troglodytes*–NCBI (GenBank: KX061886.1); and *H*. *sapiens*–NCBI (GenBank: U13369.1). (B) 3′-RACE-sequencing of 18S rRNA from cells in Fig 1 (Gal::Pno1;Gal::Dim1 cells grown in glucose to deplete endogenous Pno1 and Dim1 and supplemented with plasmids expressing Pno1 and Dim1-E85A). Read depth at each nucleotide normalized to the number of reads aligning to the 3′-end of 18S rRNA. Nucleotide positions of 18S rRNA. Above each graph is a schematic of the 18S rRNA and the ITS1 sequence above their corresponding nucleotide position and read depth. A black line indicates the 3′-end of 18S rRNA. (C) 3′-RACE-sequencing of 18S rRNA extracted from 40S ribosomes purified from cells expressing plasmid-encoded WT Pno1 and Dim1-E85A or WT Rps14 and WT Dim1. (Left) Normalized read depth upstream of the 3′-end of 18S rRNA. (Right) The fraction of reads with miscleaved 18S rRNA 3′-ends is the same in both cells. Data are the averages of 2 to 3 biological replicates, and error bars indicate SEM. N.S. not statistically significant, by unpaired *t* test. (D) Schematic representation of plasmid-encoded *Saccharomyces cerevisiae* 35S rDNA repeating unit, containing the 5′-External Transcribed Spacer (ETS), 18S rDNA, Internal Transcribed Spacer 1 (ITS1), 5.8S rDNA, ITS2, 25S rDNA, and the 3′-ETS. The only difference between the 6 constructs is the 3′-end of the 18S rDNA sequence either ending at the canonical 3′-end (WT) or containing a deletion of 1 to 4 nucleotides at the 3′-end (18S-1, 18S-2, 18S-3, and 18S-4), indicated by a black zigzag line. These plasmids encode WT or “miscleaved” rRNAs either under a constitutively active GPD promoter or a galactose-inducible, glucose-repressible Gal7 promoter. Raw numerical values to make panels B and C are available as Supporting information under **S8 Data**.
(EPS)

S3 Fig Differences in accumulation of wt 18S and miscleaved 18S rRNAs are due to differences in decay not transcription. (A) Pulse-chase analysis of rRNA stability. Tagged wt 18S or miscleaved 18S-2 and 18S-4 rRNAs were expressed in the background of endogenous wt ribosomes (BY4741 cells). At t = 0, rRNA transcription was turned off by switching to dextrose media, and the stability of the remaining tagged 18S rRNA was measured using northern blotting (left). (Right) Quantification of 2 biological replicates of data as on the left. Curve fitting to a single exponential decay model gives rate constants for 18S rRNA decay of 0.0037 min^-1^, 0.016 min^-1^, and 0.010 min^-1^ for wt 18S, 18S-2, and 18S-4, respectively. (B) Total RNA northern blots of plasmid-encoded WT 18S (top) or miscleaved 18S-2 rRNA (bottom) over time. In each panel, NOY504 cells were first grown to mid-log phase at 30°C (first time point), and then switched to 37°C (subsequent samples were taken over time). (C) Plasmid-encoded WT 18S (open symbols) or miscleaved 18S-2 rRNA (closed symbols) levels were normalized to U2 snRNA at each time point and plotted against the number of cell doublings following the switch in temperature. The time point before the switch in growth conditions is indicated as 0 doublings. Raw numerical values to make panels A and B are available as Supporting information under **S9 Data**.
(EPS)

S4 Fig Ribosome runoff gradients show slower transit times for 18–4 relative to wt ribosomes. Gradients for ribosome runoff experiments. NOY504 cells expressing either wt (A) or 18S-4 (B) rRNAs were harvested at different indicated time points after addition of lactimidomycin (LTM) to block translation initiation. The volume under the peaks for 80S ribosomes (indicated) and in the polysomes was calculated and plotted in Fig 4A.
(EPS)

S5 Fig Partial overlap between RQC and degradation of dysfunction rRNA. (A) Deletion of Dom34 leads to the accumulation of dysfunctional unprocessed pre-18S rRNA (20S Tag). Quantification of 20S rRNA levels from the northern blots in S6B Fig and additional replicates. 20S/U2 ratios from cells expressing each rRNA variant were normalized to the 20S/U2 ratios from cells expressing WT 18S. (B) Quantification of 18S tag levels in northern blots in S6B Fig and additional replicates. Plasmid-encoded 18S rRNA were normalized to U2 snRNA. 18S/U2 ratios from cells expressing each rRNA variant were normalized to the 18S/U2 ratios from cells expressing WT 18S for each cell background (fold change = 1). Data are the averages of 2–6 biological replicates, and error bars indicate SEM. N.S. not statistically significant, *p_adj_ < 0.05, **p_adj_ < 0.01, ***p_adj_ < 0.001, ****p_adj_ < 0.0001, by one-way ANOVA (Dunnett’s multiple comparison’s test) compared to BY4741 for each 18S rRNA variant. (C) Levels of 18S-4 miscleaved 18S rRNA (or all rRNAs for Rps3) relative to wt rRNA in cells lacking components of the RQC machinery. Plasmid-encoded 18S rRNA was normalized to U2 snRNA. 18S/U2 ratios from cells expressing miscleaved rRNA were normalized to the 18S/U2 ratios from cells expressing WT 18S for each cell background (fold change = 1). Data are the averages of 2–6 biological replicates, and error bars indicate SEM. N.S. not statistically significant, *p_adj_ < 0.05, **p_adj_ < 0.01, ***p_adj_ < 0.001, ****p_adj_ < 0.0001, by *t* test (wt and mutant Rps20, BY4741, and Cue2), one-way ANOVA (Dunnett’s multiple comparison’s test, for Rqt components) or two-way ANOVA wt and mutant Rps3. (D) Changes in doubling time of wild-type cells (BY4741), cells lacking Asc1 (ΔAsc1), or cells lacking Dom34 (ΔDom34), each supplemented with plasmids encoding WT 18S, an empty vector, or miscleaved 18S rRNAs under a Gal7 promoter grown at 30°C. ΔAsc1 cells were supplemented with a plasmid encoding U24 snRNA, normally encoded in the *ASC1* intron. Doubling times were normalized to WT 18S for each cell background (fold change = 1). Data are the averages of 12–19 biological replicates, and error bars indicate SEM. N.S. not statistically significant, *p_adj_ < 0.05, **p_adj_ < 0.01, by one-way ANOVA (Dunnett’s multiple comparisons test) compared to wild-type cells for each 18S rRNA variant. (E) Changes in growth of yeast cells expressing either WT 18S rRNA or miscleaved 18S-4 rRNA (or all rRNAs for Rps3 wt and K212R). Doubling times were normalized to WT 18S for each cell background. Data are the averages of 8–11 biological replicates, and error bars indicate SEM. N.S. not statistically significant, *p_adj_ < 0.05, **p_adj_ < 0.01, by *t* test (wt and mutant Rps20, BY4741 and Cue2), one-way ANOVA (Dunnett’s multiple comparison’s test, for Rqt components) or two-way ANOVA wt and mutant Rps3. Raw numerical values to make this figure available as Supporting information under **S10 Data**.
(EPS)

S6 Fig Northern blots of 18S rRNAs. Northern blots of total RNA from cells quantified in Figs 5 and S5.
(TIF)

S7 Fig Pno1-KKKF mutant that bypasses Rio1 stabilizes miscleaved 18S rRNAs. (A) Structure of the pre-40S ribosome bound to Nob1 (dark green) and Pno1 (purple). Human Nob1-bound pre-40S (PDB: 6ZXE, [30]) was aligned to yeast Pno1-bound pre-40S (PDB: 6FAI, [33]) using the MatchMaker tool in UCSF Chimera [113], using Pno1 as the reference for the alignment. The 3′-end of the yeast 18S rRNA is shown in black. The rest of the ribosome is hidden for clarity. Mutations in yeast Pno1, Pno1-KKKF (K208E/K211E/K213E/F214A), are shown as dark blue spheres. (B) 3′-RACE-sequencing of 18S rRNAs extracted from 40S ribosomal subunits purified from cells expressing Dim1-E85A and either Pno1 or Pno1-KKKF from Fig 6A. Left: Read depth at each nucleotide normalized to the number of reads aligning to the 3′-end of 18S rRNA, downstream of the cleavage site. Above the graph is a schematic of the 18S rRNA and the ITS1 sequence above their corresponding nucleotide position and read depth. A black line indicates the D cleavage site that forms the 3′-end of 18S rRNA. Right: The fraction of reads miscleaved after each of the first 5 nucleotides in ITS1. “Total” represents the cumulative miscleavage from 18S+1 to 18S+5, respectively. Data are the average of 8 biological replicates, and error bars indicate SEM (error bars are too small to be seen for many data points). N.S. not statistically significant, by ratio paired *t* test comparing miscleavage in Pno1 and Pno1-KKKF for each nucleotide. Pno1 and Pno1-KKKF samples grown and analyzed on the same day were considered paired replicates. (C) Data from Fig 6A (left) and S7B Fig (right) shown as the fold change in miscleavage at the indicated position in cells expressing Pno1-KKKF relative to miscleavage in cells expressing Pno1. Same statistical analyses as performed in Figs 6A and S7B, respectively. (D) 3′-RACE-sequencing of 25S rRNA extracted from 60S ribosomal subunits purified from Gal::Pno1; Gal::Dim1 cells depleted of endogenous Pno1 and Dim1 by growth in glucose and supplemented with plasmids expressing Dim1-E85A and either Pno1 or Pno1-KKKF. Read depth at each nucleotide position normalized to the total number of reads aligning to the 3′-end of 25S rRNA, shown within 25S rRNA upstream of the 3′-end (left) or within the 3′-ETS downstream of the 3′-end of 25S rRNA (middle). Nucleotide position in 25S rRNA is indicated. The fraction of reads miscleaved at the 3′-end of 25S rRNA, which is not affected by Pno1-KKKF (right). Data are the averages of 2 biological replicates, and error bars indicate SEM. N.S. not statistically significant, by unpaired *t* test. Raw numerical values to make panels B, C, and D are available as Supporting information under **S11 Data**.
(TIF)

S8 Fig Rio1 expression from the Cyc1 and Cup1 promoters. Western blots show that the Cyc1 promoter expresses Rio1 to near endogenous levels, while the Cup1 promoter overexpresses Rio1. Proteins from NOY504 or BY4741 cells have endogenous Rio1 expression. NOY504;Gal::Rio1 cells were depleted of endogenous Rio1 by growth in glucose dropout media and were supplemented with either an empty vector or plasmids encoding Rio1 from the Cyc1 or Cup1 promoters. Media was supplemented with 10 μm CuSO_4_ to activate the Cup1 promoter. All cells were grown at 30°C. Samples on the right were run on the same western blot and samples on the left were run on the same western blot. The order of each was edited for clarity.
(EPS)

S9 Fig Nob1 recognizes the rRNA structure at the +1A. (A) Top view of late pre-40S ribosomes with Nob1 (in red) bound. The ITS1 sequence is shown in blue, and the location of the Nob1 cleavage site, which produces the 18S 3′-end is indicated. Adapted from PDBID 6ZXF. (B) Zoom-in of the structure in A, highlighting interactions of Nob1 (in red), with 18S rRNA. For clarity, 40S ribosomal proteins are not shown. The +1A residue is indicated, together with the main chain carbonyl and the side-chain hydroxyl of threonine 237. (C). Overlay of 18S rRNA in late pre-40S ribosomes with (PDBID 6ZXE, shown in light blue) and without Pno1 (PDBID 6ZXF, shown in gray). ITS1 sequences are in dark blue in both cases. The structure shows how the active site is over the −1 residue in the earlier (+Pno1) structure.
(EPS)

S10 Fig Cancer cells overexpress RIOK1 more often than other related assembly factors. RNAseq data from The Cancer Genome Atlas (TCGA) Research Network showing mRNA expression levels of RIOK1, RIOK2, or hCINAP (human Fap7) relative to the averaged ribosomal protein (RP) mRNA expression level in cancer tissue normalized to patient-matched normal tissue. The number of patients with the indicated mRNA expression levels is plotted binned in increments. Raw numerical values to make this figure available as Supporting information under **S12 Data**.
(EPS)

S1 Table Yeast strains used in this work. (DOCX)

S2 Table Plasmids used in this work. (DOCX)

S3 Table Oligonucleotides used in this work. (DOCX)

S1 Data Raw numerical values to make the panels in Fig 1. (XLSX)

S2 Data Raw numerical values to make panels A, B, and D in Fig 2. (XLSX)

S3 Data Raw numerical values to make panels A, B, and D in Fig 3. (XLSX)

S4 Data Raw numerical values to make panels A, C, and D in Fig 4. (XLSX)

S5 Data Raw numerical values to make panels in Fig 5. (XLSX)

S6 Data Raw numerical values to make panels A, B, and D in Fig 6. (XLSX)

S7 Data Raw numerical values to make panels B, D, and E in Fig 7. (XLSX)

S8 Data Raw numerical values to make S2B and S2C Fig. (XLSX)

S9 Data Raw numerical values to make S3A and S3C Fig. (XLSX)

S10 Data Raw numerical values to make S5 Fig. (XLSX)

S11 Data Raw numerical values to make S7B and S7C Fig. (XLSX)

S12 Data Raw numerical values to make S10 Fig. (XLSX)

S1 Raw Images Raw images. (PDF)

## Abbreviations

DRD: dysfunctional rRNA decay
ITS1: Internal Transcribed Spacer 1
LTM: lactimidomycin
MBP: maltose-binding protein
NGD: no-go-decay
NRD: nonfunctional rRNA decay
QC: quality control
RP: ribosomal protein
RQC: ribosome-associated quality control
rRNA: ribosomal RNA
UMI: unique molecular identifier
WT: wild type
