# Supplementary figures and images for: The kinase Rio1 and a ribosome collision-dependent decay pathway survey the integrity of 18S rRNA cleavage
Source: PLoS Biol. 2024 Apr 25;22(4):e3001767. doi: 10.1371/journal.pbio.3001767 (PMC11045238; doi:10.1371/journal.pbio.3001767)

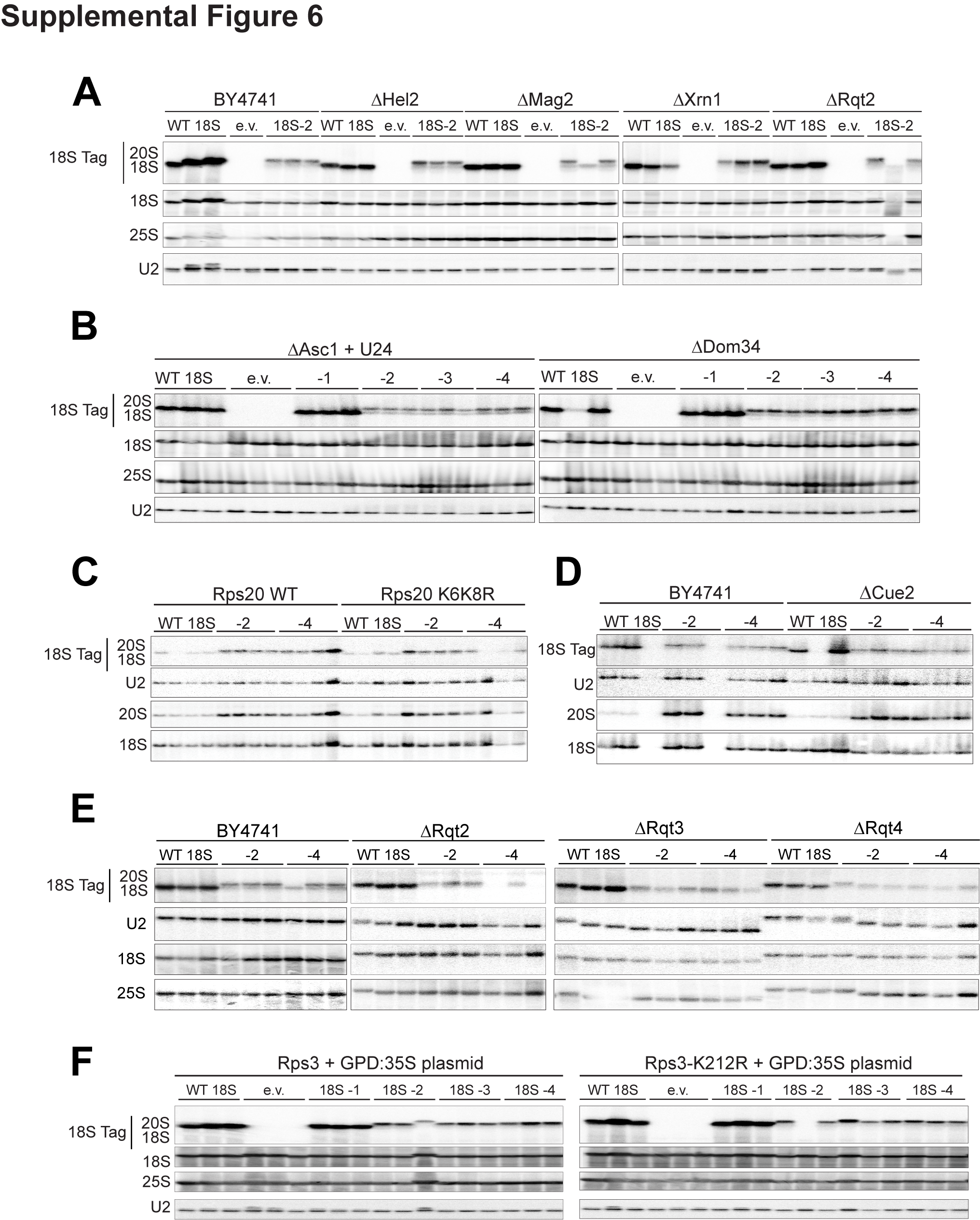

Supplement: S6 Fig — Northern blots of total RNA from cells quantified in Figs 5 and S5. (TIF) [file pbio.3001767.s006.tif]

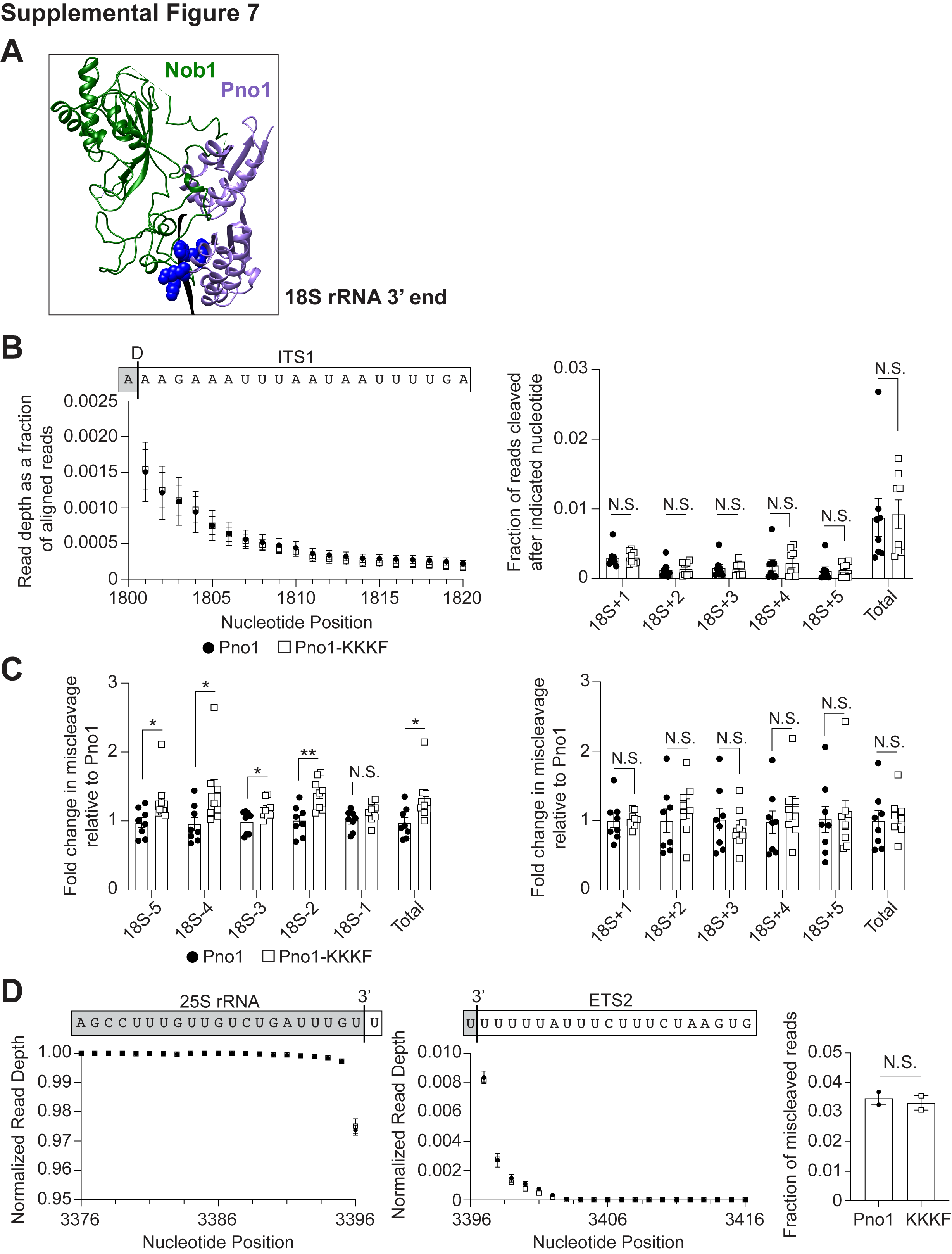

Supplement: S7 Fig — (A) Structure of the pre-40S ribosome bound to Nob1 (dark green) and Pno1 (purple). Human Nob1-bound pre-40S (PDB: 6ZXE, [30]) was aligned to yeast Pno1-bound pre-40S (PDB: 6FAI, [33]) using the MatchMaker tool in UCSF Chimera [113], using Pno1 as the reference for the alignment. The 3′-end of the yeast 18S rRNA is shown in black. The rest of the ribosome is hidden for clarity. Mutations in yeast Pno1, Pno1-KKKF (K208E/K211E/K213E/F214A), are shown as dark blue spheres. (B) 3′-RACE-sequencing of 18S rRNAs extracted from 40S ribosomal subunits purified from cells expressing Dim1-E85A and either Pno1 or Pno1-KKKF from Fig 6A. Left: Read depth at each nucleotide normalized to the number of reads aligning to the 3′-end of 18S rRNA, downstream of the cleavage site. Above the graph is a schematic of the 18S rRNA and the ITS1 sequence above their corresponding nucleotide position and read depth. A black line indicates the D cleavage site that forms the 3′-end of 18S rRNA. Right: The fraction of reads miscleaved after each of the first 5 nucleotides in ITS1. “Total” represents the cumulative miscleavage from 18S+1 to 18S+5, respectively. Data are the average of 8 biological replicates, and error bars indicate SEM (error bars are too small to be seen for many data points). N.S. not statistically significant, by ratio paired t test comparing miscleavage in Pno1 and Pno1-KKKF for each nucleotide. Pno1 and Pno1-KKKF samples grown and analyzed on the same day were considered paired replicates. (C) Data from Fig 6A (left) and S7B Fig (right) shown as the fold change in miscleavage at the indicated position in cells expressing Pno1-KKKF relative to miscleavage in cells expressing Pno1. Same statistical analyses as performed in Figs 6A and S7B, respectively. (D) 3′-RACE-sequencing of 25S rRNA extracted from 60S ribosomal subunits purified from Gal::Pno1; Gal::Dim1 cells depleted of endogenous Pno1 and Dim1 by growth in glucose and supplemented with plasmids expressing Dim1-E85A and [file pbio.3001767.s007.tif]
