# Supplementary material for: The kinase Rio1 and a ribosome collision-dependent decay pathway survey the integrity of 18S rRNA cleavage
Source: PLoS Biol. 2024 Apr 25;22(4):e3001767. doi: 10.1371/journal.pbio.3001767 (PMC11045238; doi:10.1371/journal.pbio.3001767)
Supplement: S1 Table — (DOCX) [file pbio.3001767.s011.docx]

**Table S1. Yeast strains used in this work.**

| **Strain** | **Description** | **Background** | **Genotype** | **Reference** |
| --- | --- | --- | --- | --- |
| yKK200 | BY4741 | BY4741 | *MATα his3Δ1 leu2Δ0 met15Δ0 ura3Δ0* | Euroscarf |
| yKK30 | NOY504 | W303A | *MATα Rpa12::leu2 leu2-3 ade2-1 ura3-1 his3-11 trp1-1 can1-100* | [1] |
| yKK487 | Gal::Pno1 | BY4741 | *MATα KanMX6::pGAL1-Pno1 his3Δ1 leu2Δ0 met15Δ0 ura3Δ0* | [2] |
| yKK1188 | Gal::Pno1; Gal::Dim1 | BY4741 | *MATα KanMX6::pGAL1-Pno1 NatMX6::pGAL1-Dim1 his3Δ1 leu2Δ0 met15Δ0 ura3Δ0* | This work |
| yKK988 | Gal::Pno1; Gal::Rio1 | BY4741 | *MATα KanMX6::pGAL1-Pno1 NatMX6::pGAL1-Rio1 his3Δ1 leu2Δ0 met15Δ0 ura3Δ0* | [3] |
| yKK479 | ∆Asc1 | BY4741 | *MATα Asc1::KanMX6 his3Δ1 leu2Δ0 met15Δ0 ura3Δ0* | GE Dharmacon |
| yKK395 | ∆Dom34 | BY4741 | *MATα Dom34::KanMX6 his3Δ1 leu2Δ0 met15Δ0 ura3Δ0* | GE Dharmacon |
| yKK1362 | ∆Hel2 | BY4741 | *MATα Hel2::KanMX6 his3Δ1 leu2Δ0 met15Δ0 ura3Δ0* | GE Dharmacon |
| yKK1584 | ∆Mag2 | BY4741 | *MATα Mag2::KanMX6 his3Δ1 leu2Δ0 met15Δ0 ura3Δ0* | GE Dharmacon |
| yKK1585 | ∆Slh1 | BY4741 | *MATα Slh1::KanMX6 his3Δ1 leu2Δ0 met15Δ0 ura3Δ0* | GE Dharmacon |
| yKK1634 | ∆Rqt3 | BY4741 | *MATα Rqt3::KanMX6 his3Δ1 leu2Δ0 met15Δ0 ura3Δ0* | GE Dharmacon |
| yKK1635 | ∆ Rqt4 | BY4741 | *MATα Rqt4::KanMX6 his3Δ1 leu2Δ0 met15Δ0 ura3Δ0* | GE Dharmacon |
| yKK1648 | ∆Cue2 | BY4741 | *MATα Cue2::KanMX6 his3Δ1 leu2Δ0 met15Δ0 ura3Δ0* | GE Dharmacon |
| yKK143 | ∆Xrn1 | BY4741 | *MATα Xrn1::KanMX6 his3Δ1 leu2Δ0 met15Δ0 ura3Δ0* | GE Dharmacon |
| yKK1649 | Mbf1_HA | BY4741 | *mbf1∆::hyg, ade2∆::HA-Mbf1::Met17* | Rachel Green lab |
| yKK730 | Gal::Rps20 | BY4741 | *MATα HygMX6::pGAL1-Rps20 his3Δ1 leu2Δ0 met15Δ0 ura3Δ0* | [4] |
| yKK493 | Gal::Rps3 | BY4741 | *MATα KanMX6::pGAL1-Rps3 his3Δ1 leu2Δ0 met15Δ0 ura3Δ0* | [4] |
| yKK1582 | Gal::Pno1; ∆Asc1 | BY4741 | *MATα KanMX6::pGAL1-Pno1 Asc1::HygMX6 his3Δ1 leu2Δ0 met15Δ0 ura3Δ0* | This work |
| yKK1550 | ∆Dom34; Gal::Pno1 | BY4741 | *MATα Dom34::KanMX6 HygMX6::pGAL1-Pno1 his3Δ1 leu2Δ0 met15Δ0 ura3Δ0* | This work |
| yKK1511 | NOY504; Gal::Pno1 | W303A | *MATα HygMX6::pGA1-Pno1 Rpa12::leu2 leu2-3 ade2-1 ura3-1 his3-11 trp1-1 can1-100* | This work |
| yKK1513 | NOY504; Gal::Rio1 | W303A | *MATα NatMX6::pGA1-Pno1 Rpa12::leu2 leu2-3 ade2-1 ura3-1 his3-11 trp1-1 can1-100* | This work |

**References**

1. Nogi Y, Yano R, Dodd J, Carles C, Nomura M. Gene RRN4 in Saccharomyces cerevisiae encodes the A12.2 subunit of RNA polymerase I and is essential only at high temperatures. Mol Cell Biol. 1993;13(1):114-22. PubMed PMID: 8417319.

2. Woolls HA, Lamanna AC, Karbstein K. The Roles of Dim2 in Ribosome Assembly. J Biol Chem. 2011;286: 2578-86.

3. Parker MD, Collins JC, Korona B, Ghalei H, Karbstein K. A kinase-dependent checkpoint prevents escape of immature ribosomes into the translating pool. PLoS Biol. 2019;17(12):e3000329. Epub 2019/12/14. doi: 10.1371/journal.pbio.3000329. PubMed PMID: 31834877; PubMed Central PMCID: PMCPMC6934326.

4. Huang H, Ghalei H, Karbstein K. Quality control of 40S ribosome head assembly ensures scanning competence. The Journal of cell biology. 2020;219(11). doi: 10.1083/jcb.202004161. PubMed PMID: 33007085; PubMed Central PMCID: PMCPMC7534925.
