# Supplementary material for: The kinase Rio1 and a ribosome collision-dependent decay pathway survey the integrity of 18S rRNA cleavage
Source: PLoS Biol. 2024 Apr 25;22(4):e3001767. doi: 10.1371/journal.pbio.3001767 (PMC11045238; doi:10.1371/journal.pbio.3001767)
Supplement: S2 Table — (DOCX) [file pbio.3001767.s012.docx]

**Table S2. Plasmids used in this work.**

| **Plasmid** | **Description** | **Northern Tag** | **Backbone** | **Reference** |
| --- | --- | --- | --- | --- |
| pKK30660 | GPD::35S rDNA (WT 18S) | 18S + 25S Tag | pRS426 | This work |
| pKK30661 | GPD::35S rDNA (18S -1) | 18S + 25S Tag | pRS426 | This work |
| pKK30662 | GPD::35S rDNA (18S -2) | 18S + 25S Tag | pRS426 | This work |
| pKK30663 | GPD::35S rDNA (18S -3) | 18S + 25S Tag | pRS426 | This work |
| pKK30664 | GPD::35S rDNA (18S -4) | 18S + 25S Tag | pRS426 | This work |
| pKK30665 | GPD::35S rDNA (18S -5) | 18S + 25S Tag | pRS426 | This work |
| pKK30905 | GPD::35S rDNA (MS2:WT 18S) | 18S + 25S Tag | pRS426 | This work |
| pKK30906 | GPD::35S rDNA (MS2:18S -1) | 18S + 25S Tag | pRS426 | This work |
| pKK30907 | GPD::35S rDNA (MS2:18S -2) | 18S + 25S Tag | pRS426 | This work |
| pKK30908 | GPD::35S rDNA (MS2:18S -3) | 18S + 25S Tag | pRS426 | This work |
| pKK30909 | GPD::35S rDNA (MS2:18S -4) | 18S + 25S Tag | pRS426 | This work |
| pKK30910 | GPD::35S rDNA (MS2:18S -5) | 18S + 25S Tag | pRS426 | This work |
| pKK3589 | Gal7::35S rDNA (MS2:WT 18S) | 18S Tag | pWL109 | This work |
| pKK30849 | Gal7::35S rDNA (MS2:18S -1) | 18S Tag | pWL109 | This work |
| pKK30852 | Gal7::35S rDNA (MS2:18S -2) | 18S Tag | pWL109 | This work |
| pKK30855 | Gal7::35S rDNA (MS2:18S -3) | 18S Tag | pWL109 | This work |
| pKK30858 | Gal7::35S rDNA (MS2:18S -4) | 18S Tag | pWL109 | This work |
| pKK30861 | Gal7::35S rDNA (MS2:18S -5) | 18S Tag | pWL109 | This work |
| pKK3270 | TEF::Pno1 | NA | pRS416 | [1] |
| pKK3823 | TEF::Pno1-KKKF | NA | pRS416 | [2] |
| pKK30197 | TEF::Pno1 | NA | pRS413 | This work |
| pKK30198 | TEF::Pno1-KKKF | NA | pRS413 | This work |
| pKK30224 | TEF::Pno1 | NA | pRS415 | This work |
| pKK30225 | TEF::Pno1-KKKF | NA | pRS415 | This work |
| pKK30156 | TEF::Rio1 | NA | pRS415 | [3] |
| pKK30911 | CYC1::Rio1 | NA | pRS413 | This work |
| pKK30028 | CUP1::Rio1 | NA | pRS423 | [3] |
| pKK3522 | ACT/U24 | NA | pRS413 | [4] |
| pKK3565 | TEF::Dim1-E85A | NA | pRS416 | [5] |
| pKK3594 | TEF-Rps3 | NA | pRS415 | This work |
| pKK31026 | TEF-Rps3_K212R | NA | pRS415 | This work |
| pKK3888 | TEF-Rps20 | NA | pRS413 | This work |
| pKK31133 | TEF-Rps20_K6R/K8R | NA | pRS413 | This work |
| pKK1181 | MBP-Rio1 | NA | pSV272 | [3] |
| pKK246 | H44-A2 | NA | pUC19 | [6] |
| pKK239 | H44-D | NA | pUC19 | [6] |
| pKK1278 | MBP-MS2 | NA | pMal-c | [7] |

NA = not applicable

**References:**

1. Woolls HA, Lamanna AC, Karbstein K. The Roles of Dim2 in Ribosome Assembly. J Biol Chem. 2011;286: 2578-86.

2. Johnson MC, Ghalei H, Doxtader KA, Karbstein K, Stroupe ME. Structural Heterogeneity in Pre-40S Ribosomes. Structure. 2017;25(2):329-40. doi: 10.1016/j.str.2016.12.011. PubMed PMID: 28111018; PubMed Central PMCID: PMCPMC5314460.

3. Parker MD, Collins JC, Korona B, Ghalei H, Karbstein K. A kinase-dependent checkpoint prevents escape of immature ribosomes into the translating pool. PLoS Biol. 2019;17(12):e3000329. Epub 2019/12/14. doi: 10.1371/journal.pbio.3000329. PubMed PMID: 31834877; PubMed Central PMCID: PMCPMC6934326.

4. Collins JC, Ghalei H, Doherty JR, Huang H, Culver RN, Karbstein K. Ribosome biogenesis factor Ltv1 chaperones the assembly of the small subunit head. The Journal of cell biology. 2018;217(12):4141-54. doi: 10.1083/jcb.201804163. PubMed PMID: 30348748.

5. Ghalei H, Trepreau J, Collins JC, Bhaskaran H, Strunk BS, Karbstein K. The ATPase Fap7 Tests the Ability to Carry Out Translocation-like Conformational Changes and Releases Dim1 during 40S Ribosome Maturation. Molecular cell. 2017;67(6):990-1000 e3. doi: 10.1016/j.molcel.2017.08.007. PubMed PMID: 28890337.

6. Lamanna AC, Karbstein K. Nob1 binds the single-stranded cleavage site D at the 3'-end of 18S rRNA with its PIN domain. Proc Natl Acad Sci U S A. 2009;106(34):14259-64. Epub 2009/08/27. doi: 0905403106 [pii]

10.1073/pnas.0905403106. PubMed PMID: 19706509; PubMed Central PMCID: PMC2732849.

7. Huang H, Parker M, Karbstein K. The modifying enzyme Tsr3 establishes the hierarchy of Rio kinase binding in 40S ribosome assembly. Rna. 2022;28(4):568-82. Epub 20220114. doi: 10.1261/rna.078994.121. PubMed PMID: 35031584; PubMed Central PMCID: PMCPMC8925970.
