# Supplementary material for: The kinase Rio1 and a ribosome collision-dependent decay pathway survey the integrity of 18S rRNA cleavage
Source: PLoS Biol. 2024 Apr 25;22(4):e3001767. doi: 10.1371/journal.pbio.3001767 (PMC11045238; doi:10.1371/journal.pbio.3001767)
Supplement: S3 Table — (DOCX) [file pbio.3001767.s013.docx]

**Table S3: Oligonucleotides used in this work.**

| **DNA oligonucleotide** | **Sequence (5’ to 3’)** | **Usage** |
| --- | --- | --- |
| 18S | CATGGCTTAATCTTTGAGAC | Northern blot |
| 18S TAG | CGCCGAGGATCCAACTAGGGGGCT | Northern blot |
| 25S | GCCCGTTCCCTTGGCTGTG | Northern blot |
| 25S TAG | GGGCAGGCTGCAGCTTCCTACCAG | Northern blot |
| 20S | GCTCTCATGCTCTTGCC | Northern blot |
| Probe 003  (Between A2 and A3) | TGTTACCTCTGGGCCC | Northern blot |
| U2 | ACAGGCGTCAACCATCAAGT | Northern blot |
| UMI Linker | Phos-CTGTGGAATTCTCGGGTNNNNNNNN  NNNNNNNNNNNNNNNNNCTGTAGGCACCA  TCAAT-SpC3 | 18S rRNA 3’-RACE |
| Linker 1 RT primer | ATTGATGGTGCCTACAG | 18S and 25S rRNA 3’-RACE |
| P5 adapter_Linker 1 reverse primer | CTACACGACGCTCTTCCGATCTATTGATGG  TGCCTACAG | 18S rRNA 3’-RACE |
| P7 adapter_18S 62nt  forward primer | CAGACGTGTGCTCTTCCGATCTCATTTAGA  GGAACTAAAAGTC | 18S rRNA 3’-RACE |
| Universal miRNA Cloning Linker (NEB) | rAppCTGTAGGCACCATCAAT–NH2 | 25S rRNA 3’-RACE |
| P7 adapter_Linker 1 reverse primer | CAGACGTGTGCTCTTCCGATCTATTGATGG  TGCCTACAG | 25S rRNA 3’-RACE |
| P5 adapter_25S 47nt forward primer | CTACACGACGCTCTTCCGATCTCTTGTTGT  TACGATCTGC | 25S rRNA 3’-RACE |
| T7 promoter_H44 forward primer | GCTCGGTACCCGGGGATCTAATACGACTC  ACTATAGGCCGCCCGTCGCTA | transcription template |
| D site reverse primer | mUmAATGATCCTTCCGCAGGTTCAC | transcription template |
| D+3 reverse primer | mCmUTTAATGATCCTTCCGCAGGTTCAC | transcription template |
| D-4 reverse primer | mGmATCCTTCCGCAGGTTCACCTACGG | transcription template |

Phos: Phosphorylation

N nucleotides: random nucleotides (A, C, T, or G)

SpC3: C3 Spacer phosphoamidite

UMI: unique molecular identifier

RT: reverse transcription

m: 2’-O-methylated RNA nucleotide
