# Supplementary material for: The kinase Rio1 and a ribosome collision-dependent decay pathway survey the integrity of 18S rRNA cleavage
Source: PLoS Biol. 2024 Apr 25;22(4):e3001767. doi: 10.1371/journal.pbio.3001767 (PMC11045238; doi:10.1371/journal.pbio.3001767)
Supplement: S1 Raw Images — (PDF) [file pbio.3001767.s026.pdf]

Figure 2B

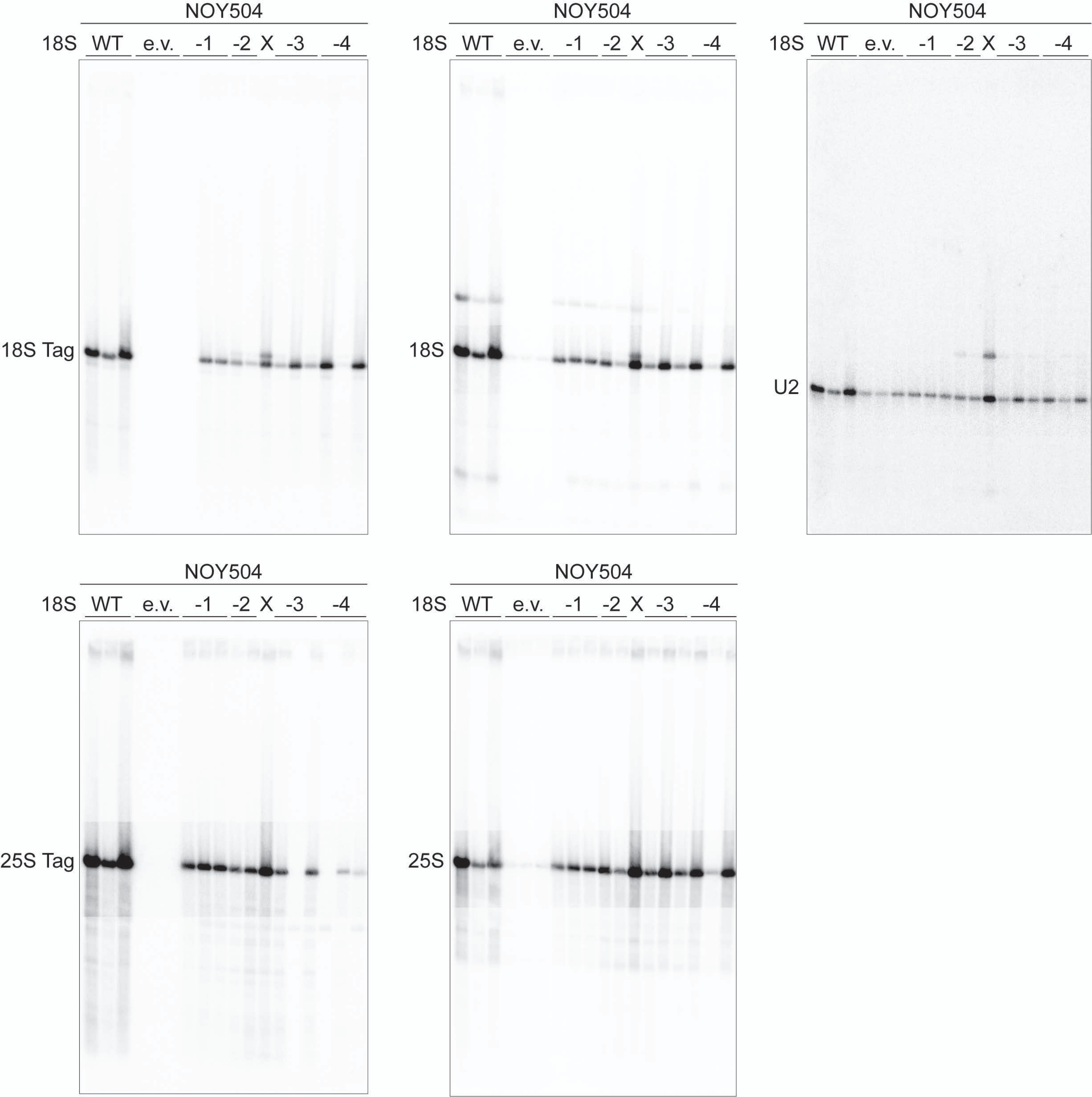
NOY504
18S WT e.v. -1 -2 X -3 -4
18S Tag
NOY504
18S WT e.v. -1 -2 X -3 -4
18S
NOY504
18S WT e.v. -1 -2 X -3 -4
U2
NOY504
18S WT e.v. -1 -2 X -3 -4
25S Tag
NOY504
18S WT e.v. -1 -2 X -3 -4
25S

Figure 2C

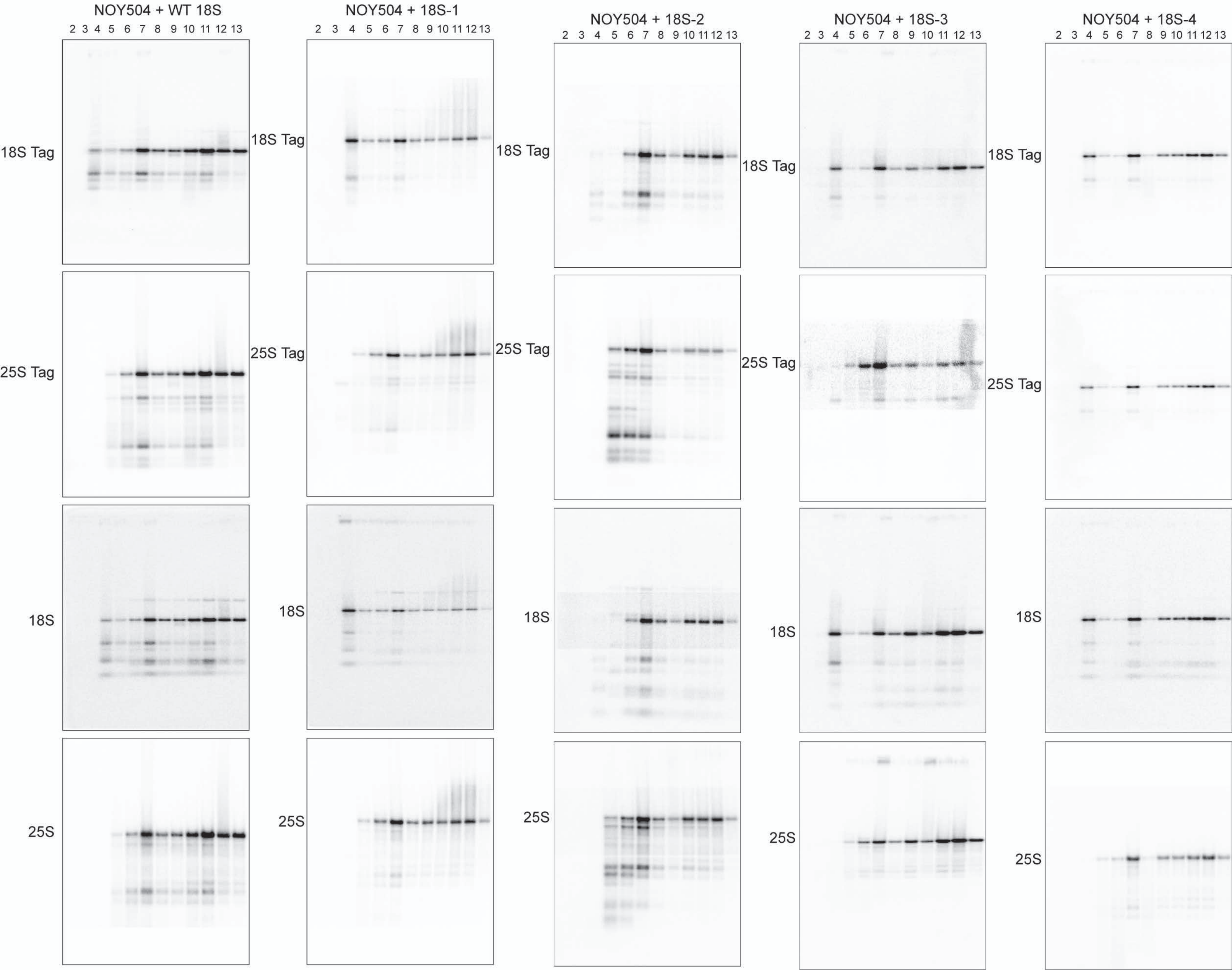
NOY504 + WT 18S
NOY504 + 18S-1
NOY504 + 18S-2
NOY504 + 18S-3
NOY504 + 18S-4
2 3 4 5 6 7 8 9 10 11 12 13
18S Tag
25S Tag
18S
25S

Figure 3B

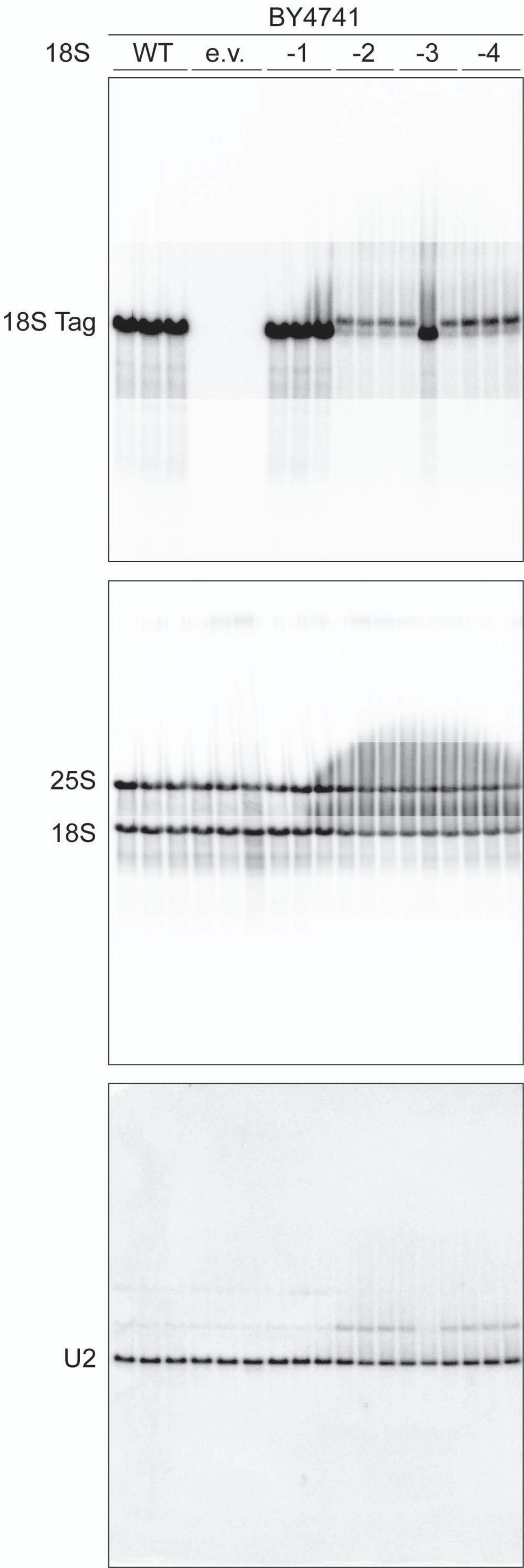

Figure 3C

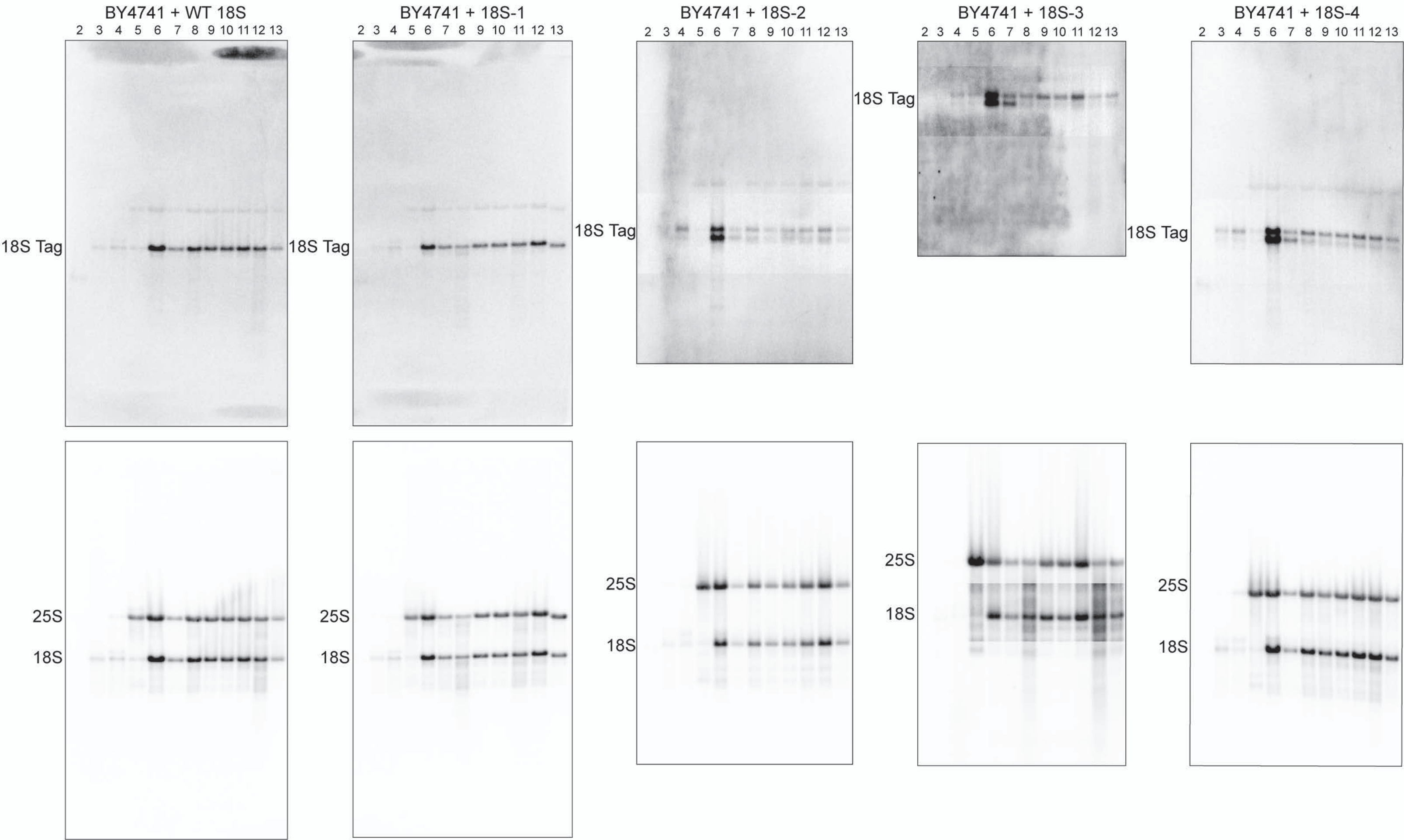
BY4741 + WT 18S
BY4741 + 18S-1
BY4741 + 18S-2
BY4741 + 18S-3
BY4741 + 18S-4
2 3 4 5 6 7 8 9 10 11 12 13
18S Tag
25S
18S

Figure 4B

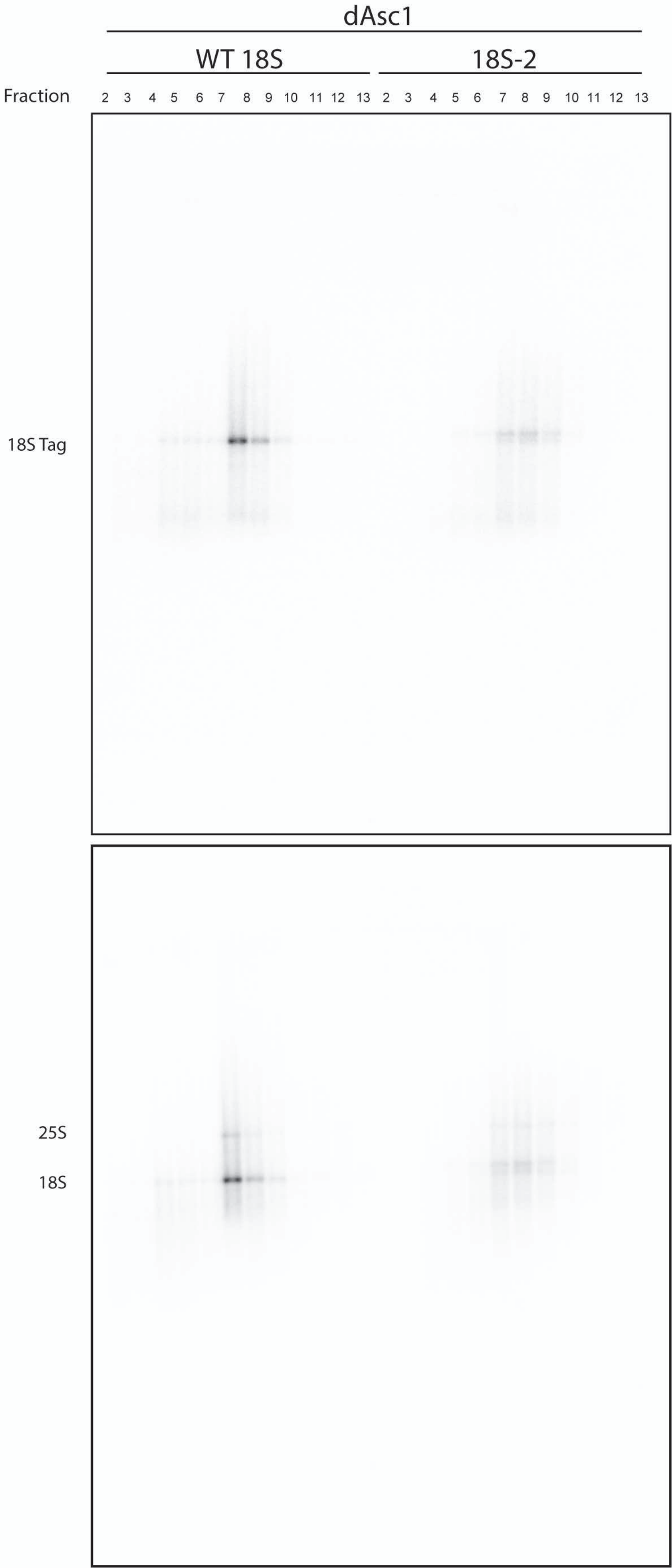
dAsc1
WT 18S
18S-2
Fraction
2 3 4 5 6 7 8 9 10 11 12 13 2 3 4 5 6 7 8 9 10 11 12 13
18S Tag
25S
18S

Figure 4D

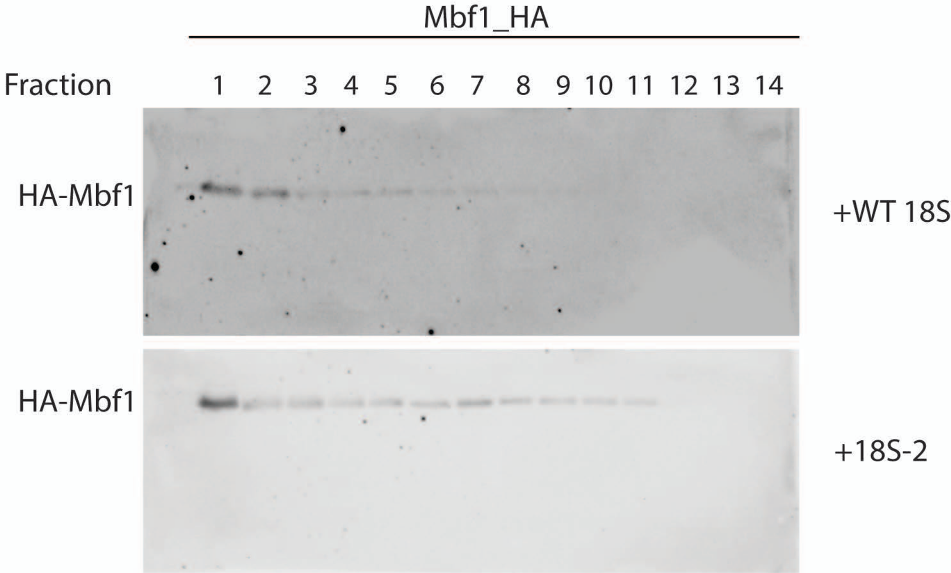
Mbf1_HA
Fraction
1 2 3 4 5 6 7 8 9 10 11 12 13 14
HA-Mbf1
+WT 18S
HA-Mbf1
+18S-2

Figure 6B

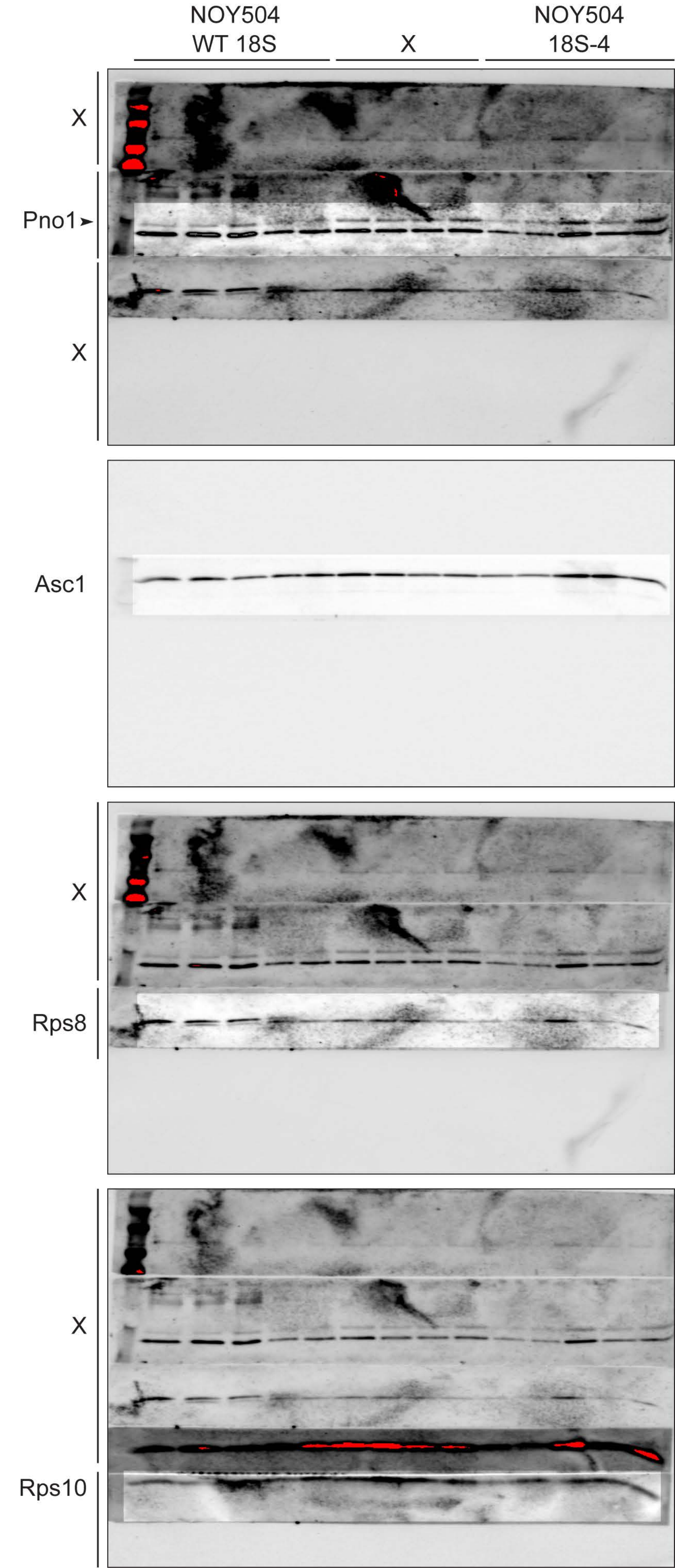

Figure 7C

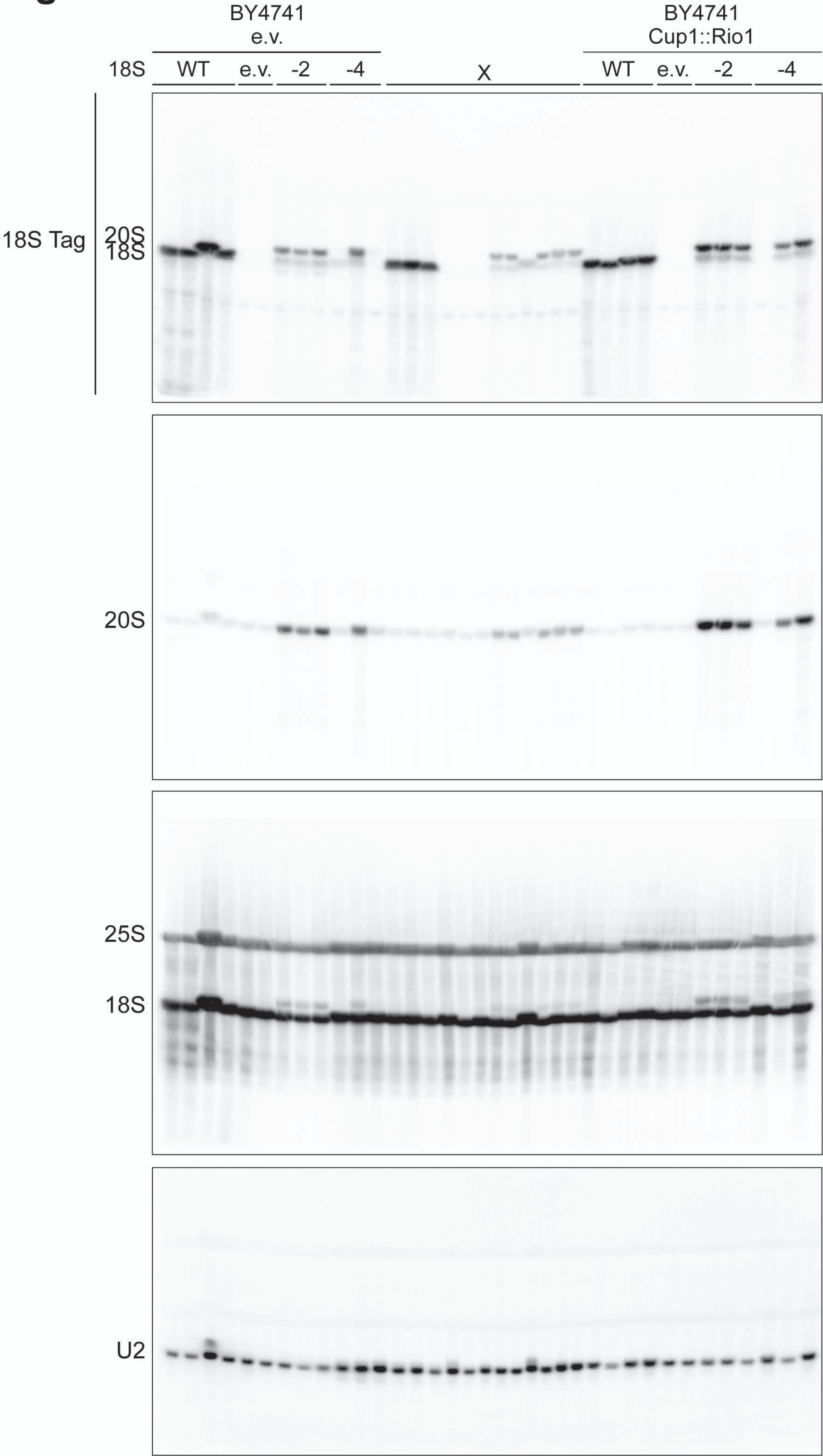
BY4741
e.v.
BY4741
Cup1::Rio1
18S
WT
e.v.
-2
-4
X
WT
e.v.
-2
-4
18S Tag
20S
18S
20S
25S
18S
U2

Figure S3A

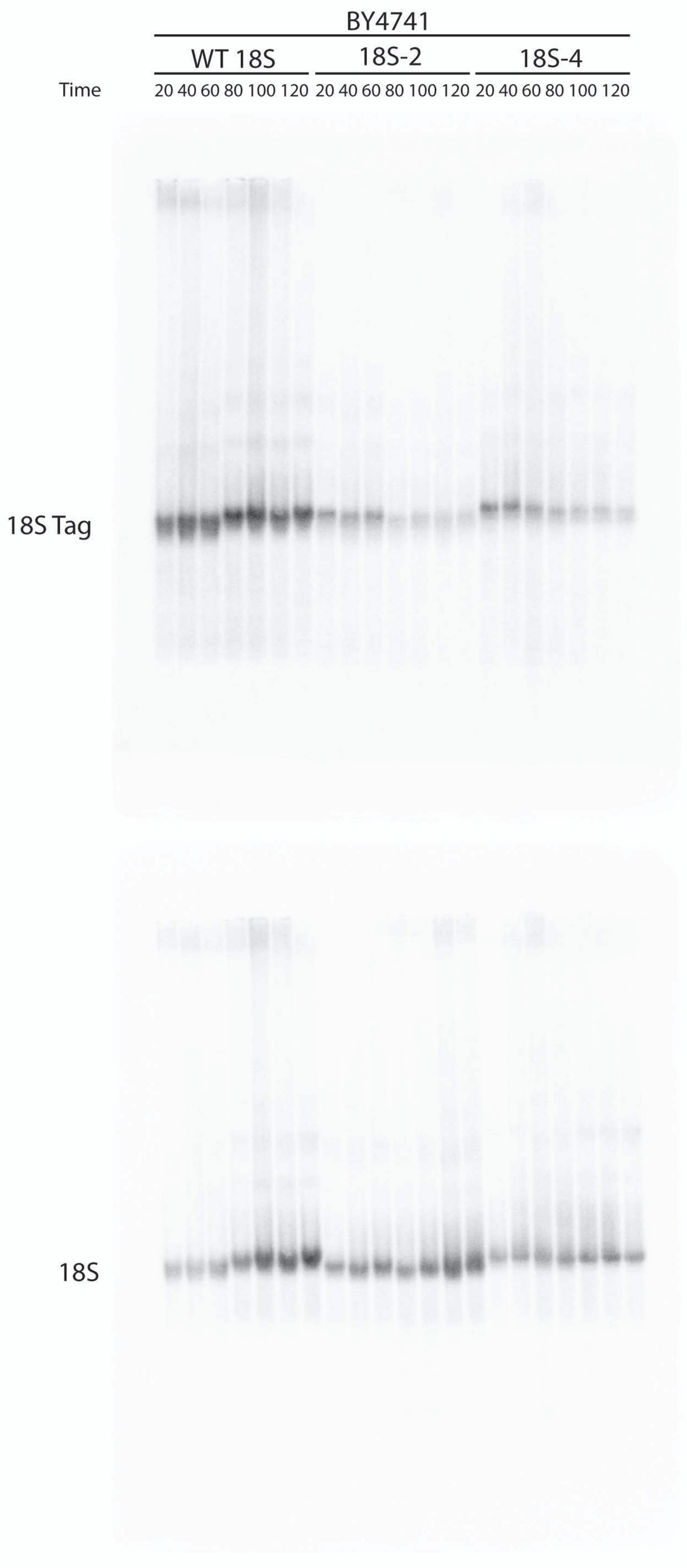
BY4741
WT 18S
18S-2
18S-4
Time
20 40 60 80 100 120 20 40 60 80 100 120 20 40 60 80 100 120
18S Tag
18S

Figure S3B

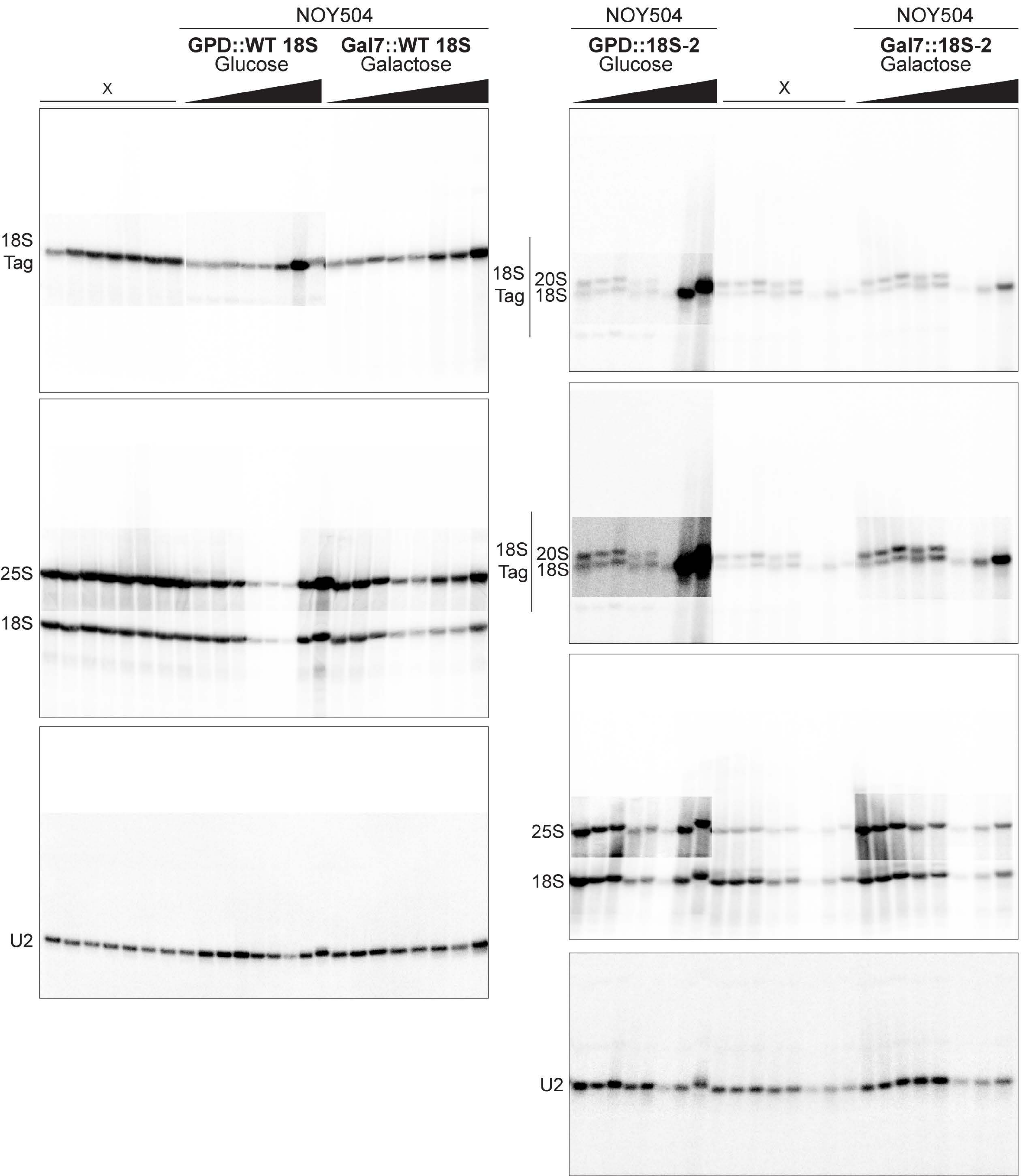

NOY504
GPD::WT 18S
Glucose
Gal7::WT 18S
Galactose
x
18S
Tag
25S
18S
U2
NOY504
GPD::18S-2
Glucose
x
NOY504
Gal7::18S-2
Galactose
18S
Tag
20S
18S
18S
Tag
20S
18S
25S
18S
U2

Figure S6A

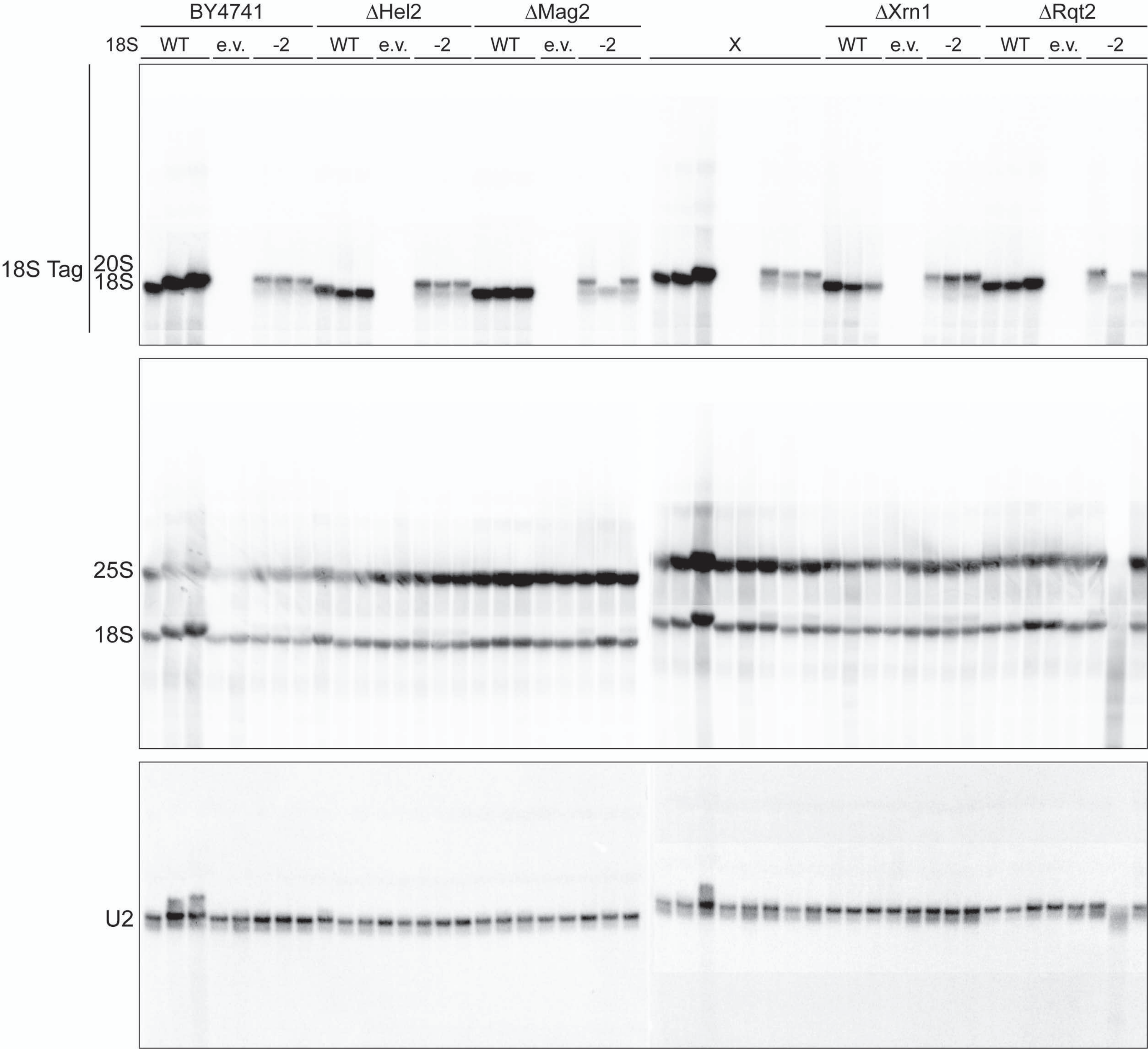
BY4741
ΔHel2
ΔMag2
ΔXrn1
ΔRqt2
18S
WT
e.v.
-2
X
18S Tag
20S
18S
25S
18S
U2

Figure S6B

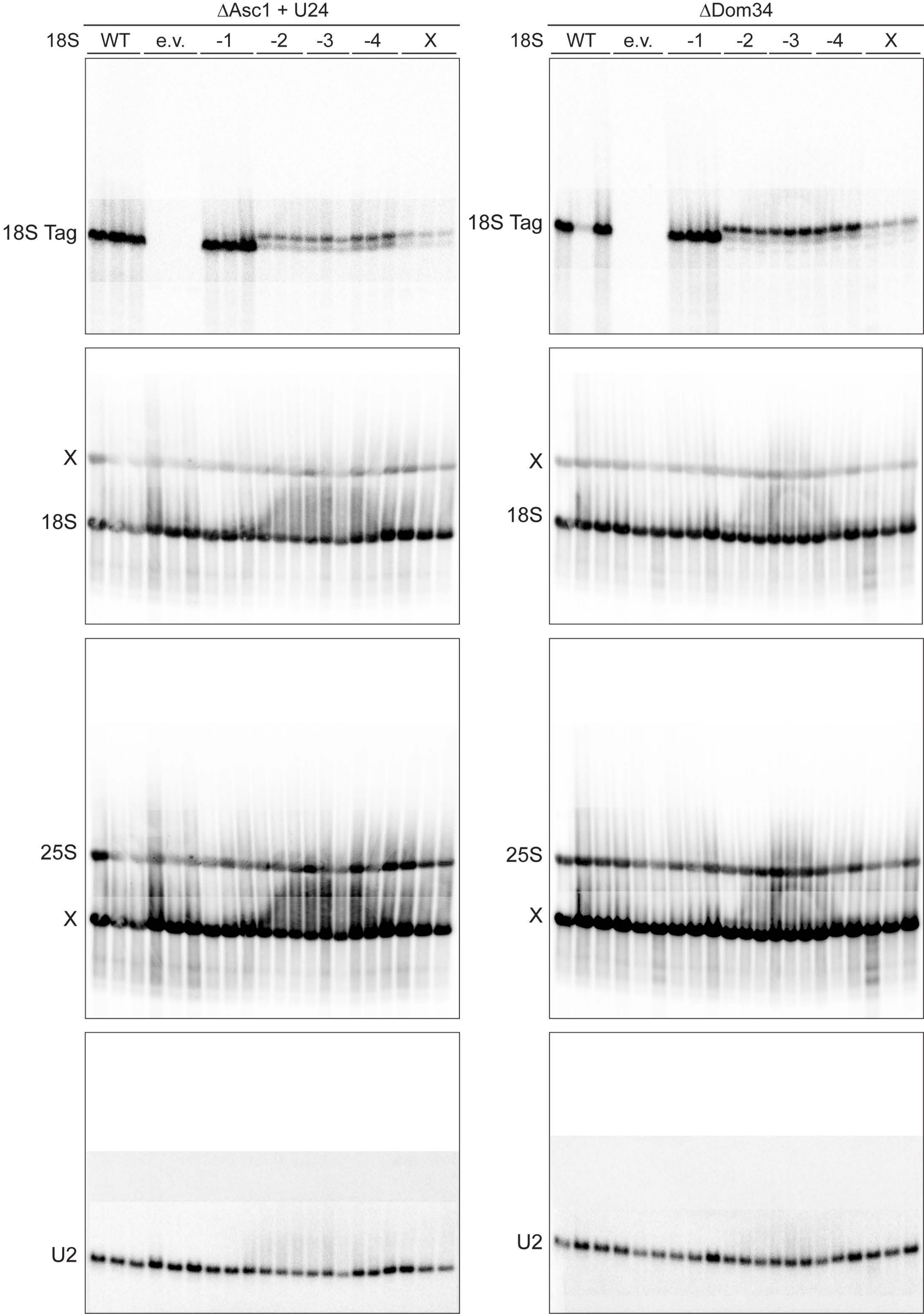

Figure S6C

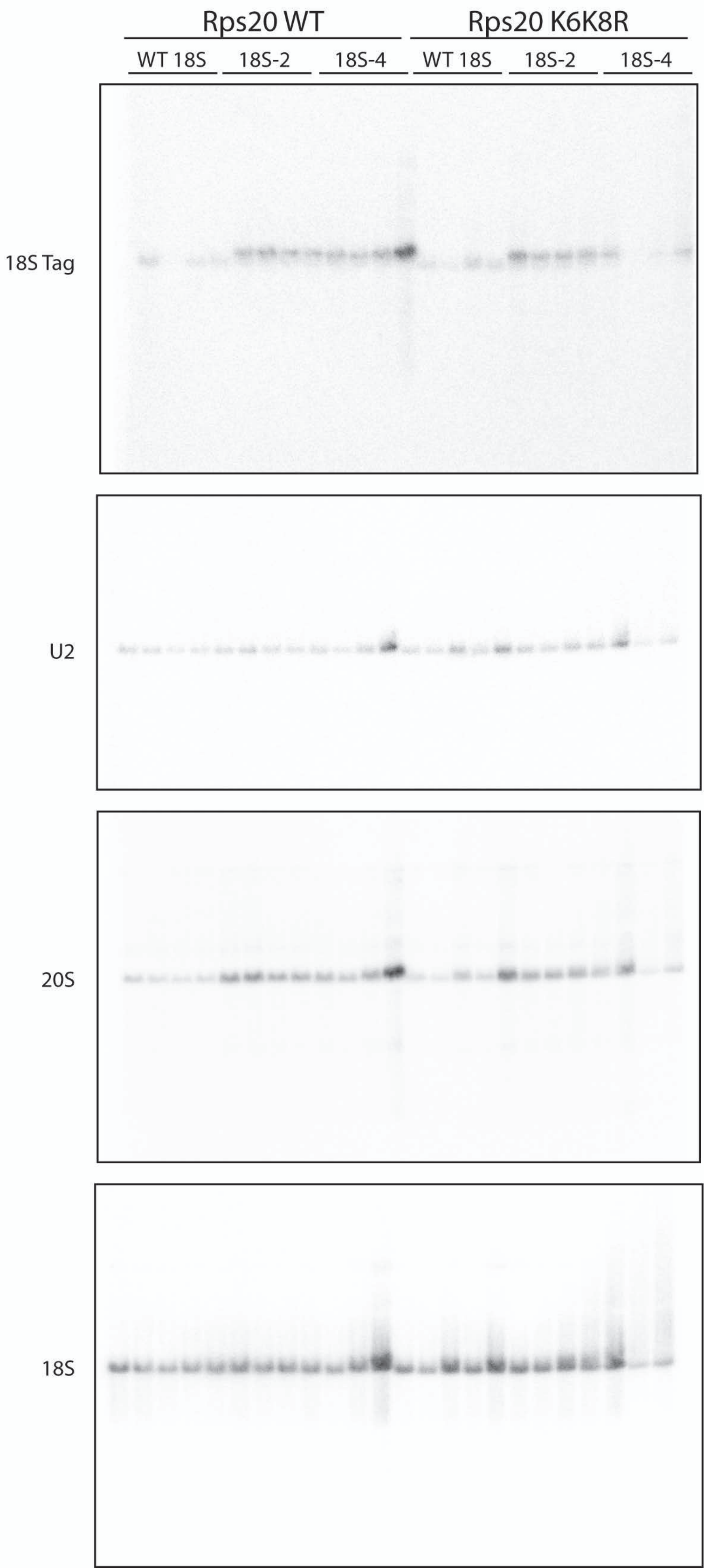
Rps20 WT
Rps20 K6K8R
WT 18S
18S-2
18S-4
WT 18S
18S-2
18S-4
18S Tag
U2
20S
18S

Figure S6D

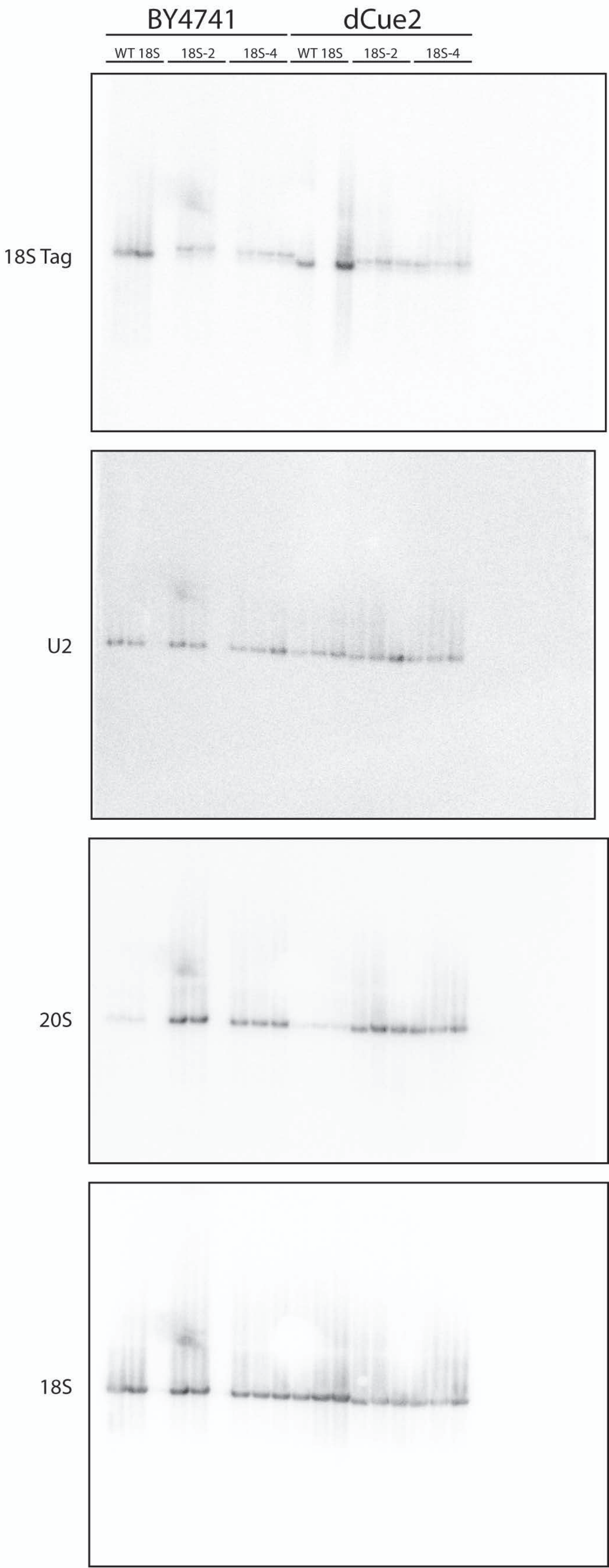
BY4741
dCue2
WT 18S
18S-2
18S-4
WT 18S
18S-2
18S-4
18S Tag
U2
20S
18S

Figure S6E

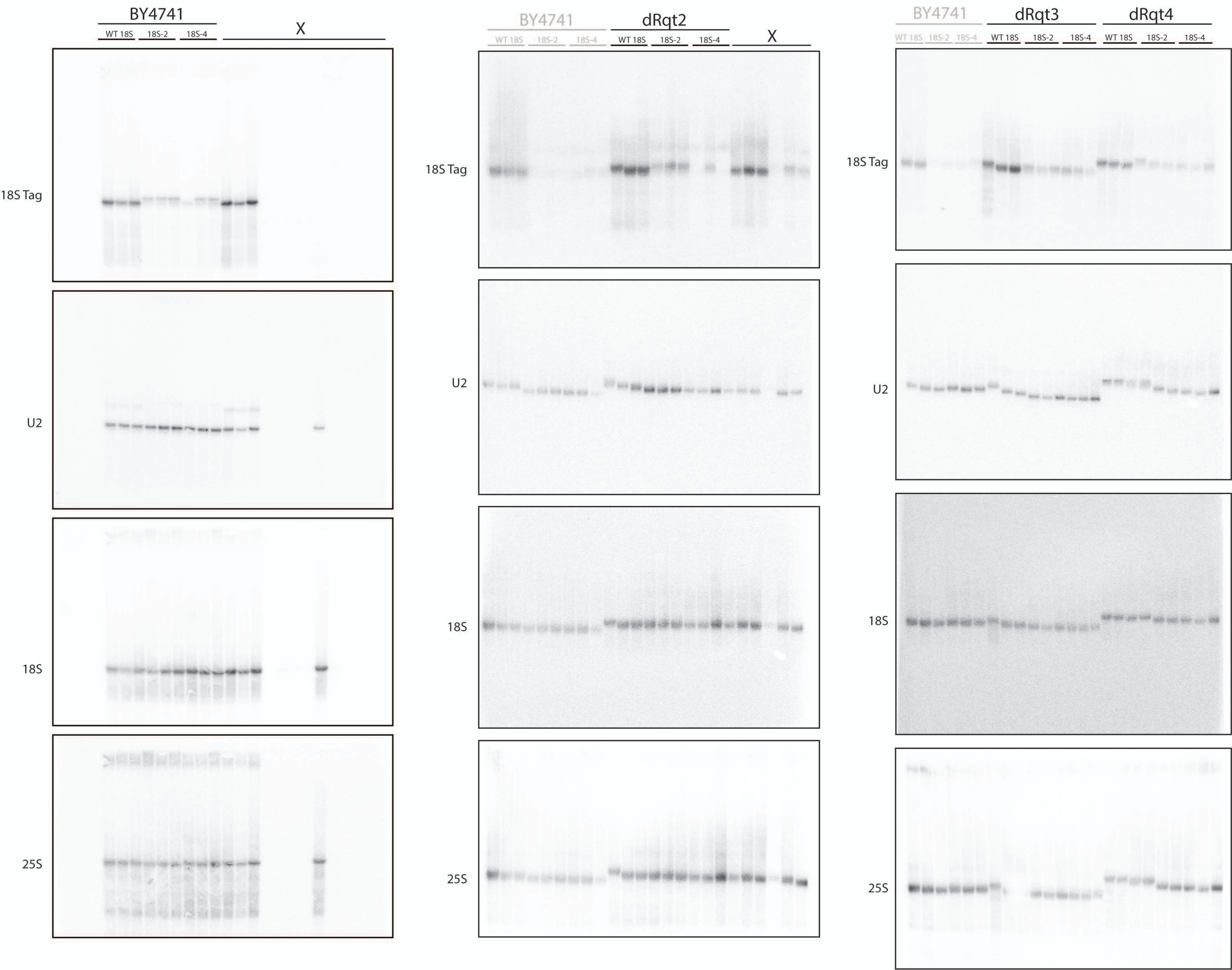

BY4741
WT 18S
18S-2
18S-4
X
18S Tag
U2
18S
25S
BY4741
dRqt2
WT 18S
18S-2
18S-4
WT 18S
18S-2
18S-4
X
18S Tag
U2
18S
25S
BY4741
dRqt3
dRqt4
WT 18S
18S-2
18S-4
WT 18S
18S-2
18S-4
WT 18S
18S-2
18S-4
18S Tag
U2
18S
25S

Figure S6F

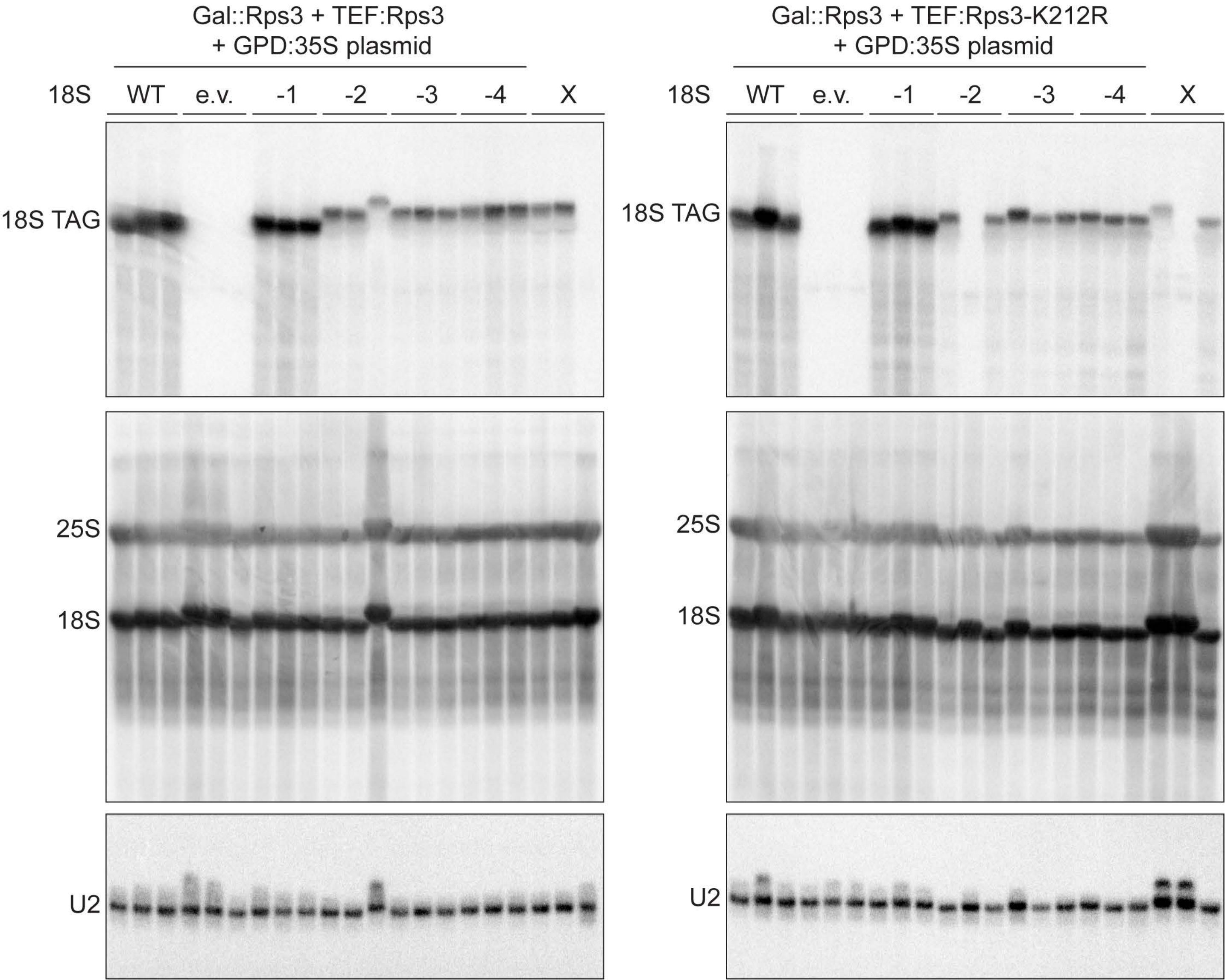

Gal::Rps3 + TEF:Rps3
+ GPD:35S plasmid
18S
WT
e.v.
-1
-2
-3
-4
X
18S TAG
25S
18S
U2
Gal::Rps3 + TEF:Rps3-K212R
+ GPD:35S plasmid
18S
WT
e.v.
-1
-2
-3
-4
X
18S TAG
25S
18S
U2

Figure S8

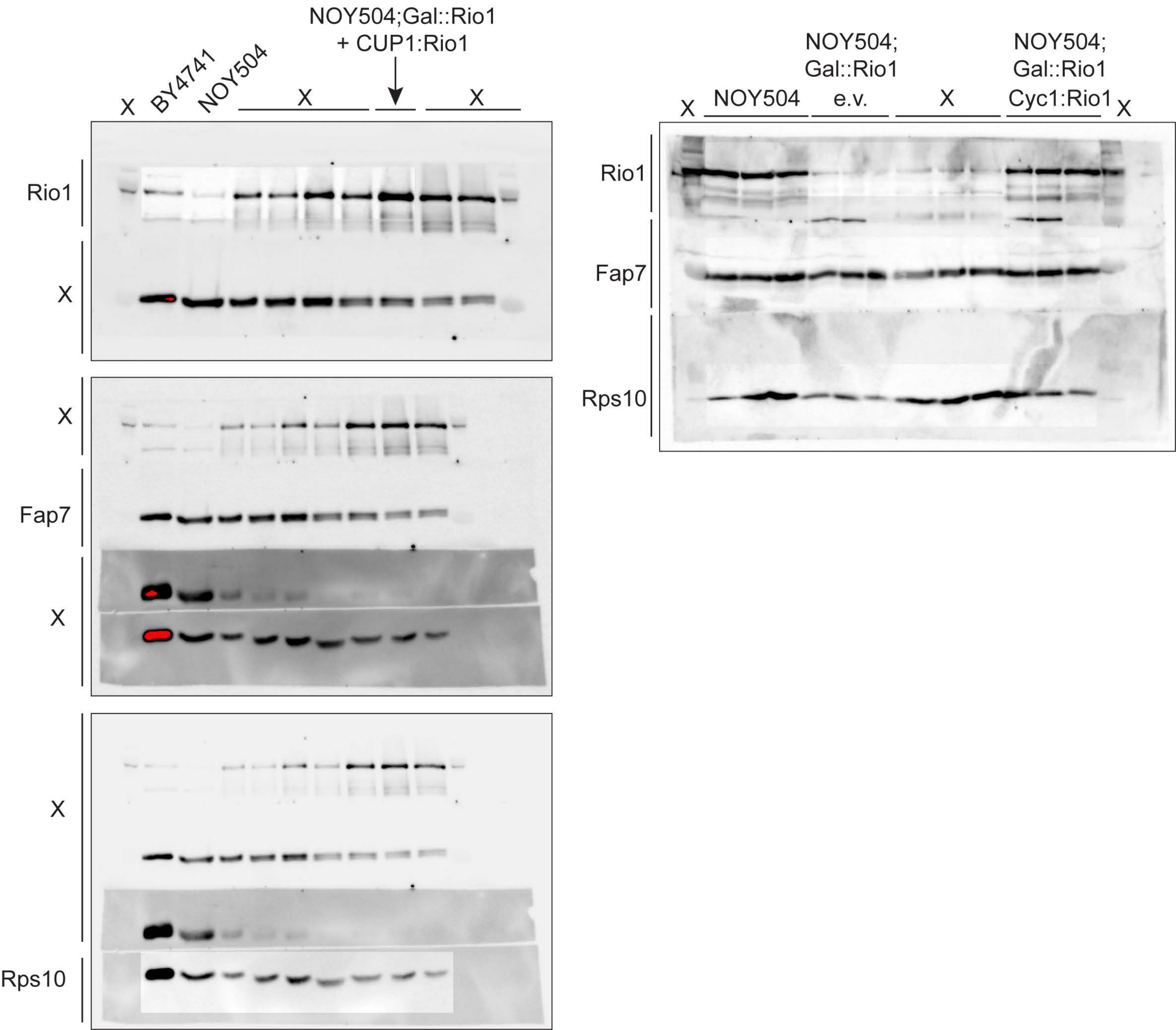
NOY504;Gal::Rio1
+ CUP1:Rio1
X
BY4741
NOY504
X
X
Rio1
X
X
Fap7
X
X
Rps10
X
NOY504
NOY504;
Gal::Rio1
e.v.
X
NOY504;
Gal::Rio1
Cyc1:Rio1
X
Rio1
Fap7
Rps10
